# The efficacy of anterior repositioning splints in the management of temporomandibular disc displacement: a systematic review and meta-analysis

**DOI:** 10.1186/s12903-025-06379-3

**Published:** 2025-07-28

**Authors:** Liujing Wang, Yanni Zhang, Haiyan Chen, Chenxi Jin, Wei Shen, Ziyuan Li, Wei Zhang, Yuxin Shi, Yiyang Hou, Xiaoxuan Li, Jing Guo

**Affiliations:** 1 Department of Stomatology, Ningbo Municipal Hospital of Traditional Chinese Medicine (TCM) Affiliated Hospital of Zhejiang Chinese Medical University, Ningbo Zhejiang, 315010 China; 2 Ningbo Stomatology Hospital, Ningbo, Zhejiang 315010 China; 3 Savaid Stomatology School, Hangzhou Medical College, Hangzhou, Zhejiang 310000 China; 4 Academician Technology Innovation Center of Ningbo Stomatology Hospitall, Ningbo, Zhejiang 315010 China; 5 Ningbo Cunji Stomatology and Otolaryngology Medical Institute, Ningbo, Zhejiang 315010 China; 6 Department of Stomatology, Women and Children’s Hospital of Ningbo University, Ningbo, Zhejiang 315300 China; 7 School of Stomatology, University of Zhejiang Chinese Medical, Hangzhou, Zhejiang 310000 China

**Keywords:** Anterior repositioning splint, Joint disc displacement, Behavioral therapy, Physical therapy, Occlusal splint

## Abstract

**Objective:**

This systematic review and meta-analysis aimed to evaluate the effectiveness of anterior repositioning splint (ARS) compared with other conservative treatments for temporomandibular joint (TMJ) disc displacement.

**Methods:**

This systematic review and meta-analysis was conducted in accordance with PRISMA guidelines. Four databases (PubMed, Web of Science, Embase, and Cochrane) were searched up to January 8, 2024, so as to identify eligible randomized controlled trials (RCTs) of ARS for the treatment of TMJ disc displacement.

**Results:**

This analysis finally included 14 studies. The research findings showed that after 3 months of treatment, ARS significantly improved VAS score compared to the physical therapy [MD: -1.90, 95%CI (-2.69, -1.11), *P* < 0.00001] and behavioral therapy [MD=-3.00, 95%CI (-4.30, -1.71), *P* < 0.05]. However, it was less effective than other occlusal splint treatments [MD: 0.71, 95%CI (0.30, 1.11), *P* = 0.0007]. In terms of pain free mouth opening, ARS significantly outperformed the physical therapy [MD = 2.83, 95%CI (1.04, 4.62), *P* = 0.002] and behavioral therapy [MD: 1.79, 95%CI (1.33, 2.26), *P* < 0.00001] after 3 months of treatment. No significant difference was observed between ARS and other occlusal splint treatments [*MD*=-0.97, 95%*CI* (-2.65, 0.71), *P* = 0.26]. Regarding maximal active mouth opening, after 3 months of treatment, ARS was significantly superior to the physical therapy [MD = 3.10, 95%CI (1.05, 5.15), *P* = 0.03] and behavioral therapy [MD: 3.19, 95%CI (2.71, 3.66), *P* < 0.00001]. There was no significant difference between ARS and other occlusal splint treatments [MD: − 1.92, 95%CI (-4.05, 0.21), *P* = 0.08]. In terms of maximal passive mouth opening, ARS was significantly more effective than physical therapy [MD: 1.91, 95%CI (0.13, 3.68), *P* = 0.04]. There was no significant difference between ARS and other occlusal splint treatments [MD: -2.25, 95%CI (-5.02, 0.53), *P* = 0.11]. For relieving jaw popping symptoms, ARS was significantly more effective than the physical therapy [RR:0.45, 95%CI (0.34, 0.58), *P* < 0.00001] and behavioral therapy [RR: 0.48, 95%CI (0.36, 0.64), *P* < 0.00001]. However, no significant difference was found between ARS and other occlusal splint treatments [RR: 1.29, 95%CI (0.83, 2.02), *P* = 0.26].

**Conclusion:**

A total of 14 studies were included in this systematic review and meta-analysis, which compared the efficacy of ARS with other conservative treatments for disc displacement. While ARS did not demonstrate a significant difference compared to physical therapy in improving TMJ pain, popping, and mandibular motor function in the initial treatment phase, it exhibited more pronounced effects than physical therapy and behavioral therapy with continued use. Splints have demonstrated significant efficacy in alleviating TMJ popping and improving mandibular movement. However, ARS has not shown superior effectiveness compared to other types of occlusal splints in alleviating pain.

**Supplementary Information:**

The online version contains supplementary material available at 10.1186/s12903-025-06379-3.

## Introduction

Temporomandibular disorders (TMD), a common group of conditions affecting the oral and maxillofacial region, is characterized by functional and/or structural impairments of the masticatory muscle system, the temporomandibular joint (TMJ), and its related anatomical components [1, 2]. The incidence rate of TMD is relatively high, and it mostly occurs in young and middle-aged individuals, especially females. According to a survey report, 60–70% of the general population have experienced TMD symptoms at least once [3]. The epidemiological survey data of various countries have shown that the incidence rate of TMD is about 10.5–54.0% [4–6], and the incidence rate is increasing year by year.

According to the new version of the Diagnostic Criteria for Temporomandibular Disorders (DC/TMD [7]) released in 2014, the clinical diagnosis of TMD is divided into two classes. Class I refers to painful diseases, including muscle pain, joint pain, and headache caused by TMD. Class II refers to joint diseases, including disc displacement with reduction, disc displacement with reduction with intermittent locking, disc displacement without reduction with limited opening, disc displacement without reduction without limited opening, degenerative joint disease, and joint subluxation. Among them, internal derangement (ID), characterized by structural disorders among the joint disc, condyle, and glenoid fossa, and primarily manifested as joint disc displacement, is the most common condition. Epidemiological studies have reported that the incidence of ID in TMD patients is as high as 36.8% [8]. The displaced joint disc hinders the normal sliding and rotation of the condyle during mandibular movement. Clinically, it manifests as a series of symptoms such as opening deviation, limited opening, joint pain, popping or noise, thus affecting the quality of life of patients and requiring active treatment interventions [9, 10].

The treatment methods for TMD include conservative treatment and non-conservative treatment. Conservative treatments include occlusal splint therapy, psychological therapy, medication therapy, physical therapy, behavioral therapy and arthrocentesis, while non-conservative treatments include arthroscopic surgery and open surgery [11]. Based on the diagnosis and severity of the disease, individualized therapy is adopted, and conservative and reversible treatment should be prioritized. More invasive arthroscopic surgery or even open surgery should only be considered after conservative management has proven ineffective [11]. In conservative treatment, occlusal splint therapy is one of the most widely applied methods. By changing the relative position of the occlusal contact between the upper and lower jaws and releasing the unfavorable load on the joint area, it can achieve neuromuscular rebalancing of the stomatognathic system, thereby managing functional disorders of the stomatognathic system [12]. According to the mechanism of action, occlusal splints are divided into stabilization splints, anterior repositioning splints, and soft occlusal splints [13]. A variety of occlusal splint types exist, each with distinct mechanisms of action, allowing for their use in addressing different clinical scenarios.

Among the various types of occlusal splints, the anterior repositioning splint (ARS) is widely used clinically. It is fabricated from hard acrylic resin and covers the maxillary or mandibular arch. Its distinct occlusal morphology (cusp-fossa contact) guides the mandible to a predetermined therapeutic position, typically anterior to the intercuspal position [12].

By repositioning the mandible, the condyle can be moved forward and downward to recapture the joint disc and reduce intra-articular pressure, thus alleviating joint popping and pain to a certain extent [14].

Despite numerous studies both domestically and internationally [1, 2, 15–18] examining its efficacy, the findings remain inconsistent. Furthermore, existing systematic reviews [19–25] primarily focused on the treatment outcomes for various types of TMD, with fewer systematic reviews specifically addressing disc displacement [26, 27]. Furthermore, design flaws existed, such as the inclusion of non-randomized controlled trials (RCTs) and the absence of comparisons with other conservative treatment modalities. Consequently, a systematic review and meta-analysis are required to rigorously assess the clinical efficacy of ARS for disc displacement versus alternative conservative approaches, with the goal of establishing evidence-based guidelines.

## Materials and methods

### Study strategy and registration

This systematic review and meta-analysis were conducted in accordance with the Preferred Reporting Items for Systematic Reviews and Meta-Analyses (PRISMA) guidelines and were registered on PROSPERO (Registration No.: CRD42024520369). PICOS strategy was adopted, which was referred to Population (P): Patients diagnosed with TMJ disc displacement; Intervention (I): The application of ARS as a treatment method; Comparison (C): The application of other treatment methods as the control group, such as physical therapy, stabilization splint therapy, and behavioral therapy; Outcome (O): Alleviation in TMJ symptoms, including pain and popping, jaw motor function, and changes in joint disc position; and Study Type (S): RCTs.

### Database and search strategy

The combination search strategy of corresponding subject words and free words was applied to conduct literature searches in four databases, including Pubmed, Web of Science, Embase, and Cochrane. The last search was on January 8, 2024. The corresponding subject words and free word included Temporomandibular joint (Joint, Temporomandibular, Joints, Temporomandibular, Temporomandibular Joints, TMJ, articulatio temporomandibularis, craniomandibular joint, jaw joint, joint, mandibular, joint, mandibulotemporal, mandible joint, mandibular joint, mandibulotemporal joint, temporo mandibular joint, temporomandibular articulation, temporomandibular joint meniscus); Anterior repositioning splint (Anterior repositioning appliances, Anterior repositioning splints, Repositioning occlusal splints, ARS, ARA); Disc displacement (disc displacements, Disk displacements, Disk displacement, Internal derangement, Anterior disc displacement with reduction, Anterior disc displacement without reduction). For each database, a comprehensive search strategy was developed, and Boolean calculation (AND, OR, NOT) was applied to combine search terms to refine the search and identify relevant articles. The reference list included in the study was manually searched to obtain other relevant articles. The specific search strategies are detailed in the Appendix.

### Inclusion criteria

According to the “Participants, Interventions, Comparisons, Outcomes, and Studies” (PICOS) guidelines, the following inclusion criteria were adopted: [1] P (Participants): Patients diagnosed with disc displacement; [2] I/C (Intervention/Comparison): Clinical studies employing ARS as a treatment modality, with other treatment approaches serving as control groups; [3] O (Outcome): a follow-up evaluation of curative effect on pain, popping, and mandibular motor function, with a follow-up duration of ≥ 3 months; [4] S (Study Type): RCTs; [5] As RDC/TMD was reported in 1992, this systematic review and meta-analysis only included studies published after 1992.

### Exclusion criteria

 [1] Trials with animals as research subjects; [2] Academic reports, case reports, reviews, comments, letters, books, conference abstracts, case series, opinion articles, technical articles, posters, and guidelines; [3] Studies that did not undergo clinical examinations during follow-up and only conducted telephone follow-up; [4] Articles published in languages other than English; [5] Clinical studies with unavailable original research data.

### Literature screening and data extraction

Literature searches were conducted from four databases, including Pubmed, Web of Science, Embase, and Cochrane. Relevant data were imported into EndNote20. Duplicates were removed, and preliminary screening was performed by reviewing article titles and abstracts. Finally, the literature was further screened by reviewing the full text. The agreement between the two researchers’ screening results was assessed using the Kappa statistic.

The data were extracted by the reviewer using an EXCEL spreadsheet from the final included literature and classified for management. The variables extracted from each study included the following: [1] Basic information of studies: the first author, year of publication, and country; [2] Information about patients: type of disc displacement, number of cases, age, gender ratio, and whether the diagnostic criteria were confirmed by MRI; [3] Interventions: intervention measures, control measures, wearing time of occlusal splints, and follow-up duration; [4] Outcome measures: Alleviation of TMJ symptoms after treatment for less than 3 months and more than 3 months, including pain VAS score, number of people with popping symptom alleviation before and after treatment, comfortable mouth opening, maximum active mouth opening, and maximum passive mouth opening [5]. Study results: The above literature search, screening, data extraction, and management were independently conducted by two reviewers, and the differences between reviewers were ultimately determined through discussion or inviting a third reviewer.

### Quality assessment and risk of bias

The included studies were all RCTs, and the quality assessment of all literature strictly followed the PRISMA principle [28]. The risk of bias assessment of the included studies was conducted using the Cochrane tool (ROB), incorporating the following seven items: random sequence generation, allocation concealment, blinding of researchers and subjects, blinding of research outcomes, integrity of outcome data, selective outcome reporting, and other sources of bias. Each item was assessed as having “high risk”, “low risk” or “unclear risk” of bias. Two reviewers independently assessed the risk of bias and quality, and any discrepancies were resolved through consensus or consultation with a third reviewer.

### Evidence quality assessment

Based on the Grading of Recommendations Assessment, Development and Evaluation (GRADE) system [29], the quality of evidence was assessed using GRADEpro GDT. The reliability of evidence may be diminished by the following five factors: study limitations, inconsistency of results, indirectness of evidence, imprecision and reporting bias. The outcomes were classified into four distinct grades: high, moderate, low, and very low.

### Data statistical analysis

The Review Manager (V5.4) software (RevMan Web, The Cochrane Collaboration, UK) was adopted for statistical analysis. For the selection of effect sizes for continuous variables, weighted mean difference (WMD) was adopted for representation when the measurement values were consistent; otherwise, standardized mean difference (SMD) was applied. The effect size of binary variables was represented by relative risk (RR) and 95% confidence interval (CI). The chi-square test was applied to evaluate the heterogeneity of the included studies, which was represented by I². The model type was determined based on the I² value. If I² was less than 50%, it was considered that the heterogeneity between studies was small. Consequently, a fixed-effects model was used. If I² was greater than 50%, it was considered that the heterogeneity between studies was significant, and a random-effects model was used. To reduce heterogeneity between studies and enhance research stability, subgroup analysis was performed on the included studies. A funnel plot was applied to assess publication bias in the included studies, and the sensitivity was adopted to evaluate the robustness of the research findings. The P-value of pooled statistics was commonly calculated using the Z-test. A P-value less than 0.05 was considered statistically significant, while a P-value more than 0.05 was considered not statistically significant.

## Results

### Study screening

Literature search was conducted in four databases, including Pubmed, Web of Science, Embase, and Cochrane, using corresponding subject words and free words. Finally, 67 articles were retrieved in PubMed, 46 in Embase, 27 in Cochrane, and 73 in Web of Science, with a total of 213 relevant articles retrieved. Additionally, 8 articles were obtained through other channels (totaling 221 articles), and 92 duplicates were excluded. After preliminary screening through reviewing the title and abstract, 96 articles were excluded. These articles involved 2 meta-analyses, 15 reviews, 3 case studies where the subjects did not meet the requirements, 12 case reports, 1 article with telephone or email follow-up, 1 animal study, 26 articles with intervention measures that did not meet the requirements, 1 conference, 4 articles with outcome measures that did not meet the requirements, 9 articles that did not meet the time requirements, 1 article with book chapters as content, 2 articles without follow-up, 11 articles with unobtainable full text, and 8 articles with inconsistent language.

**Fig. 1:**
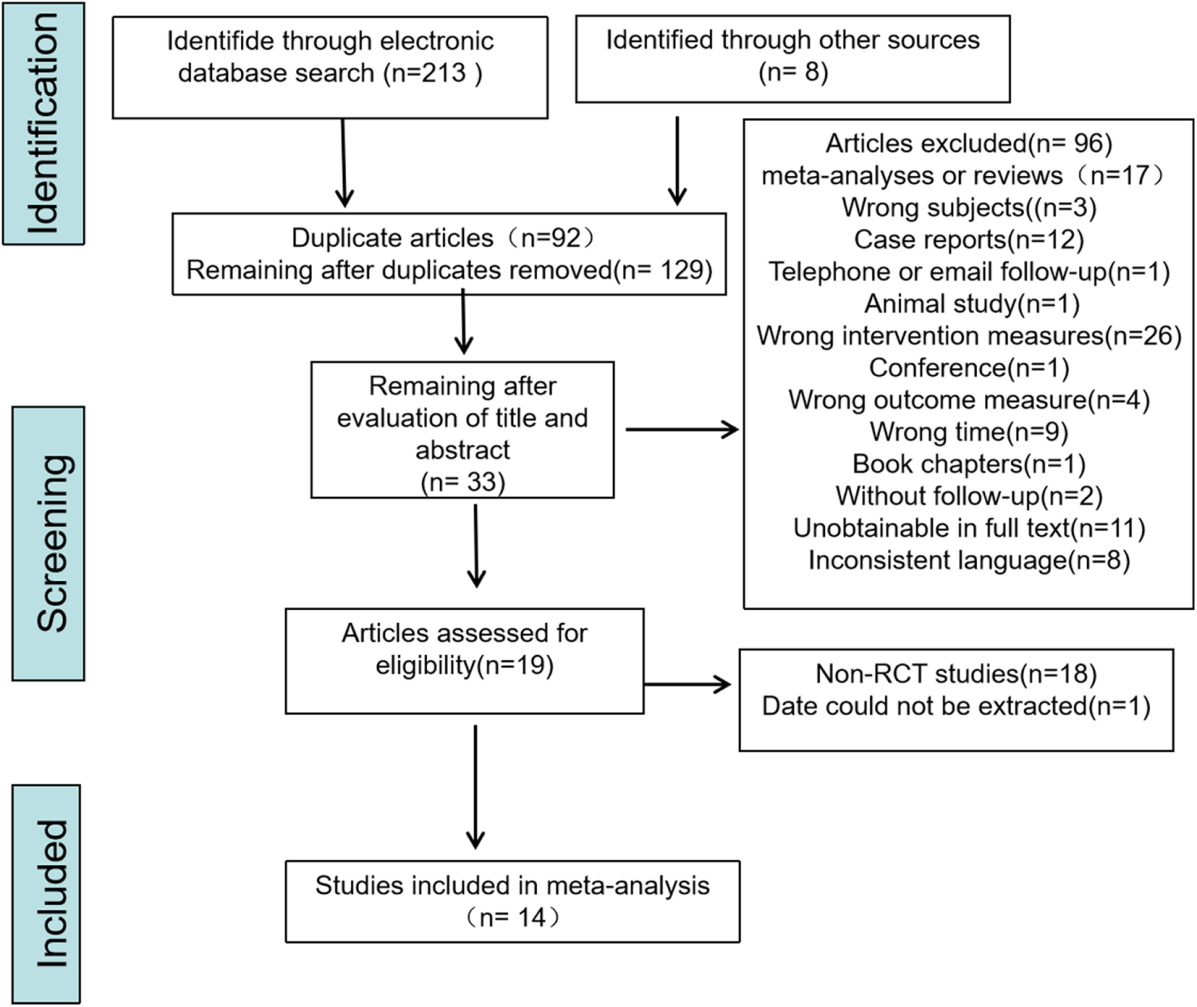
Flowchart for study screening details

After full-text reviewing of the remaining 33 articles, 18 non-RCT studies were excluded, resulting in a total of 15 articles remaining. Among them, 1 article had data that could not be extracted, and 14 articles were ultimately included [30–43]. The literature screening process is illustrated in Fig. 1. The agreement between the two researchers’ screening results was assessed using the Kappa statistic. The analysis revealed a Kappa value of 0.89, indicating good agreement.

### Basic characteristics of the included studies

The 14 studies included were published from 2004 to 2023, and the number of patients ranged from 9 to 189. The total number of patients was 1026, with Mona M.S. Fayed’s study in 2004 [39] having the smallest number of participants at only 9, and Irfan Adil Majid’s study in 2020 [40] having the largest number of participants at 189. The age range of all participants was 18 to 60 years. All 14 studies included were RCTs, of which 5 were [30, 34, 36, 39, 41] from Egypt, 2 were [32, 35] from Turkey, and the remaining 7 were from Iran [31], Germany [33], Italy [37], Brazil [38], Saudi Arabia [40], Poland [42], and India [43]. The main type of disc displacement of the participants included in the study was disk displacement with reduction (DDwR), with only one study [33] including disc displacement without reduction (DDWoR), and one study [37] not mentioning the type of disc displacement. Among the included studies, there were 7 studies [31, 32, 34, 36, 38, 40, 41]where patients mainly wore ARS at night for more than 10 h. Five studies [32, 35, 37, 39, 42] showed wearing for more than 18 h, or even 24 h a day. Additionally, 2 studies [30, 43] did not mention the duration of wearing. Among all included studies, 7 studies [30, 33, 34, 36, 37, 39, 41] had diseases diagnosed by MRI, and 7 studies [31, 32, 35, 38, 40, 42, 43] were diagnosed through clinical examination and questionnaire survey based on RDC/TMD diagnostic criteria, without undergoing MRI. In addition, there were 5 studies [30, 38, 39, 41, 43] with a follow-up duration of less than or equal to 3 months, 3 studies [31, 40, 42] of 3–6 months, and the remaining 6 studies [32–37] of greater than or equal to 6 months. The details of all included studies are shown in Tables 1 and 2.

**Table 1:** Basic characteristics of the included studies

First author	Year of publication	Country	Type of disc displacement	Total number of participants	F、M	Age	Intervention (number of participants)	MRI diagnosis	Time for wearing occlusal splints	Follow-up duration	Outcome measures
Abdel-Naser M. Emam [36]	2023	Egypt	DDwR	100	64/36	21–44	Group 1 Behavioral therapy group (23)	Yes	-	6 months	1# Vertical mouth opening 2# Lateral motion 3# Range of anterior protrusion motion
							Group 2 Low-intensity laser therapy group (25)		Twice a week		
							Group 3 ARS group (24)		At least 12 h		
							Group 4 Stable occlusal splint group (23)		At least 12 h		
Nermeen A. Rady [41]	2022	Egypt	DDwR	27	8/1	24.22 ± 2.9	Group 1 ARS group (9)	Yes	Wearing at night	3 months	1# Pain VAS score 2# Joint disc position 3# Condylar position
					8/1	23.22 ± 2.1	Group 2 Botulinum toxin type A (BTX-A) (9)		Single injection of 30 units		
					9/0	23.22 ± 2.1	Group 3 Low-intensity laser therapy group(9)		Applying 3 times a week		
Nada O. El Zawahry [30]	2021	Egypt	Chronic DDwR	30	22/8	25 ± 6.3	Group 1 ARS group (10)	Yes	Not mentioned	3 months	1# TMJ dysfunction 2# Chronic pain grading
							Group 2 Low-intensity laser therapy group (10)				
							Group 3 Placebo group (10)				
Esmail Ahmed [34]	2021	Egypt	Unilateral chronic DDwR (over 2 weeks)	100	64/36	21–44	Group 1 Behavioral therapy group (25)	Yes	6 months	6 months	1# Pain VAS score
							Group 2 Low-intensity laser therapy group (25)		Twice a week		
							Group 3 ARS therapy group (25)		12 h/day		
							Group 4 Stable occlusal splint group (25)		12 h/day		
Ravza Eraslan [35]	2021	Turkey	DDwR	80	16/4	24.15 ± 9.47	Group 1 Low-dose laser therapy group (20)	No	3 courses per week for a total of 3.5 weeks and 10 courses	6 months	1# Popping 2# Vertical mouth opening 3# Horizontal mouth opening
					16/4	30.25 ± 14.34	Group 2 Stable occlusal splint group (20)		24 h, 6 months		
					16/4	28.30 ± 9.92	Group 3 ARS group (20)		24 h, stop using after 3 months, and using a stable splint for 3 months		
					8/2	23.10 ± 5.70	Group 4 Control group of untreated patients (20)		-		
					7/3	26.70 ± 3.20	Group 5 Healthy control group of healthy subjects (20)		-		
Irfan Adil Majid [40]	2020	Saudi Arabia	DDwR	189	39/24	35 ± 14	Group 1 Behavioral therapy group (63)	No	4 months	4 months	1# Pain 2# Tenderness of masticatory muscle 3 # Comfortable mouth opening 4 # Maximum mouth opening 5 # Popping
					39/24	34 ± 14	Group 2 Physical therapy group (63)		4 months		
					38/25	36 ± 12	Group 3 ARS group (63)		Wearing for 2 h during the day + wearing at night		
Malgorzata Pihut [42]	2018	Poland	DDwR	112	40/16	31(24–45)	Group 1 ARS group (56)	No	20 h	16 weeks	1# Pain assessment
					43/13		Group 2 Biolaser group (56)		Once every other day		
Jyoti Devi [43]	2017	India	DDwR	30	4/6	27.1 ± 7.19 (18–40)	Group 1 ARS group (10)	No	Not mentioned	10 weeks	1# Comfortable mouth opening 2# Maximum mouth opening 3# Pain VAS score 4# Popping
					3/7	30.8 ± 10.36 (19–55)	Group 2 Stable occlusal splint group (10)				
					5/5	32.1 ± 15.23 (19–60)	Group 3 Soft occlusal splint group (10)				
Paulo César Rodrigues CONTI [38]	2015	Brazil	DDwR	60	58/2	38.35	Group 1 ARS group (20)	No	Wearing at night	3 months	1# Pain VAS score 2# Threshold of TMJ tenderness 3# Maximum movement range 4# Popping
						38.4	Group 2 NTI-tss (20)		Using at night		
						46	Group 3 Behavioral therapy group (20)		-		
Azam S. Madani [31]	2011	Iran	Acute DDwR < 6 months	60	15/5	27.2 ± 12.43	Group 1 ARS group (20)	No	Over 10 h per day for the first three months, and switching to a stable occlusal splint after three months	5 months	1# Pain VAS score
					19/1	23.15 ± 5.69	Group 2 Physical therapy group (20)		Once every other day , lasting for four weeks		
					19/1	22.43 ± 6.02	Group 3 Physical therapy + splint treatment group (20)		More than 10 h per day		
Hanefi KURT [32]	2011	Turkey	DDwR	105	11/9	26.55 ± 10.19	Group 1 ARS group (20)	No	Wearing at night	6 months	1# Chronic pain grading 2# Vertical mouth opening 3# Popping
					29/3	27.16 ± 8.08	Group 2 Stable occlusal splint group (32)		Wearing at night		
					17/5	26.9 ± 11.01	Group 3 Behavioral therapy group (20)		-		
Simona Tecco [37]	2006	Italy	Not mentioned	50	22/28	28.20 ± 6.5	Group 2 SVED (sagittal vertical compression device) and MORA (anterior mandibular reduction splint) therapy (20)	Yes	Wearing MORA during the day and SVED at night	6 months	1# Popping 2# Pain intensity
						27.8 ± 7.2	Group 3 Untreated patients (10)				
Marc Schmitter [33]	2005	Germany	DDWoR	74	30/6	32.5	Group 1 ARS group (36)	Yes	Wearing 18 h a day	6 months	1# Chewing pain 2# Functional pain 3# Poor chewing ability 4# Active mouth opening 5# Passive mouth opening
					35/3	42	Group 2 Stable occlusal splint group (38)				
Mona M. S. Fayed [39]	2004	Egypt	DDwR	9	2/2	24	Group 1 ARS group (4)	Yes	Wearing it all day long	3 months	1# Joint disc recapture 2# Pain degree 3# Popping
					1/4	24	Group 2 Canine protection occlusal splint group (5)				

**Table 2:** Summary of conclusions from the included studies

Literature	Conclusion
Abdel-Naser M. Emam2023 [36]	Both ARS and stabilization splints demonstrate the ability to improve maximum mouth opening in patients with DDwR.
Nermeen A. Rady2022 [41]	All groups experienced a significant reduction in pain. In symptomatic individuals with DDwR, BTX-A and LLLT demonstrated potential as effective alternatives to ARS for mitigating joint pain, popping, and improving disc position.
Nada O. El Zawahry2021 [30]	ARS, LLLT, and placebo therapy demonstrate no significant difference in improving TMJ dysfunction and chronic pain scores in patients with TMJ disc displacement.
Esmail Ahmed2021 [34]	Both stabilization splints and ARS are effective therapeutic modalities for reducing pain intensity in patients with DDwR, with stabilization splints demonstrating superior efficacy. Behavioral therapy alone is insufficient for managing chronic TMD pain. Photobiomodulation therapy is recommended as an adjunct to occlusal splint therapy for the alleviation of pain associated with TMD.
Ravza Eraslan2021 [35]	ARS demonstrate the most significant improvement in reducing popping during mouth opening in patients.
Irfan Adil Majid2020 [40]	All groups showed significant alleviation in joint pain and improvement in maximal mouth opening. The ARS group demonstrated the most marked improvements in joint popping reduction, comfortable mouth opening, and maximal mouth opening.
Malgorzata Pihut2018 [42]	ARS is an effective tool for reducing pain associated with disc displacement.
Jyoti Devi2017 [43]	ARS, stabilization splints, and soft splints result in marked amelioration of symptoms including pain, popping, and mouth opening in patients diagnosed with DDwR.
Paulo César Rodrigues CONTI2015 [38]	The concurrent application of ARS and behavioral therapy leads to a more rapid improvement in pain for patients with DDwR. The use of NTI-tss devices may exacerbate TMJ popping.
Azam S. Madani2011 [31]	ARS is considered the optimal treatment for reducing pain and popping in patients with DDwR.
Hanefi KURT2011 [32]	Behavioral therapy and occlusal splint therapy are both considered appropriate therapeutic interventions for the treatment of DDwR.
Simona Tecco2006 [37]	ARS has been demonstrated as an effective treatment modality. The implementation of a MORA splint during daytime hours and a SVED splint during nighttime hours can enhance patient comfort.
Marc Schmitter2005 [33]	Both stabilization splints and ARS demonstrate efficacy in reducing pain and increasing mandibular range of motion in patients with disc displacement. However, stabilization splints appear to exhibit superior effectiveness compared to ARS.
Mona M. S. Fayed2004 [39]	Both canine guidance splints and ARS are effective in alleviating pain and popping. Canine guidance splints offer an advantage over ARS by facilitating the restoration of the disc’s normal length and shape, concurrently promoting posterior displacement to enable recapture. Occlusal splint therapy is the treatment of choice for managing DDwR.

### Methodological quality assessment of the studies included

**Fig. 2:**
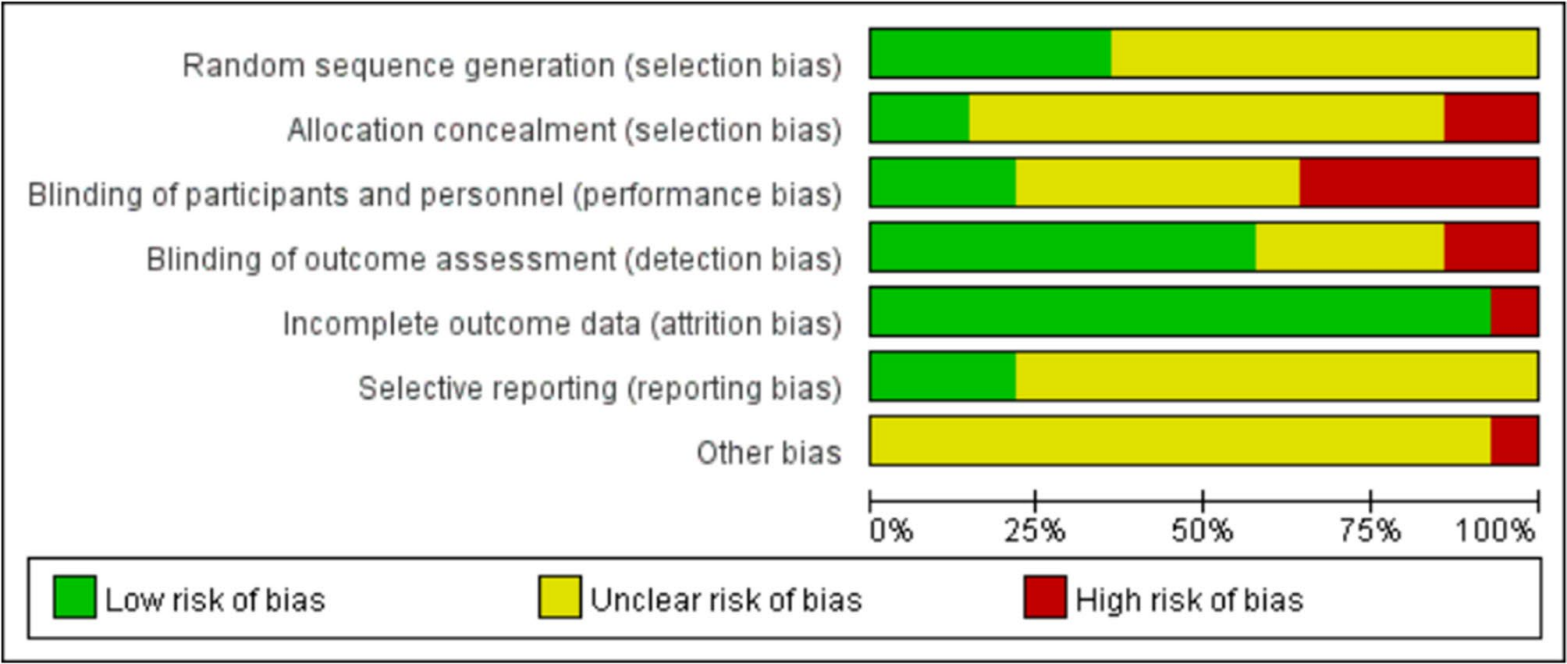
Cochrane Risk Bias Assessment (ROB)

**Fig. 3:**
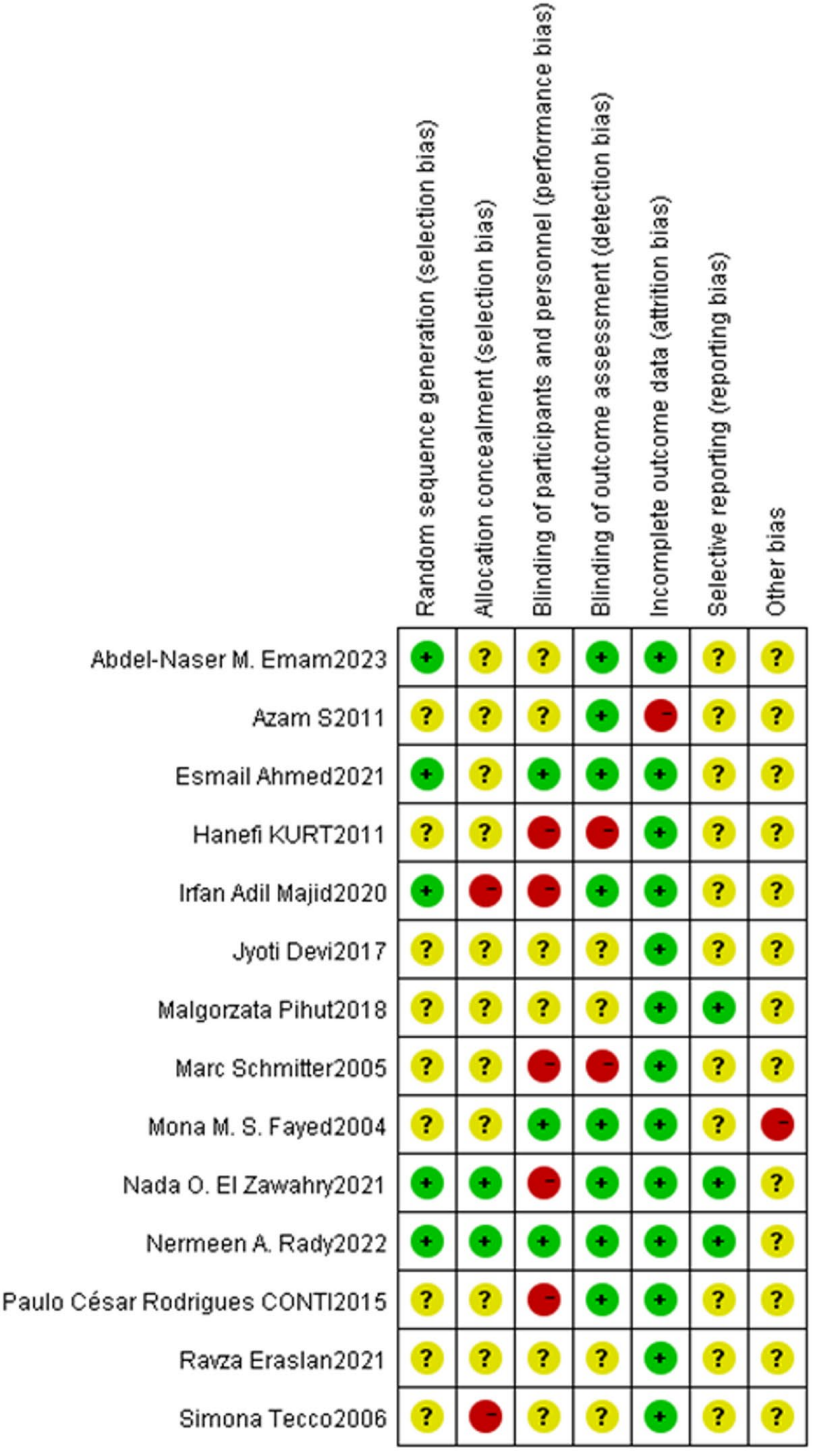
Summary of Cochrane Risk Bias Assessment

The Cochrane risk of bias assessment results showed that: (1) Generation of random sequences: 6 studies [30, 34, 36, 37, 40, 41] were considered to be at low risk, and the sequences were generated by computers. The bias risk of 8 studies [31–33, 35, 38, 39, 42, 43] was unknown, and the method of sequence generation was not described in detail. (2) Allocation concealment: Two studies [30, 41] were considered to be at low risk, and sealed envelopes were adopted, while two studies [37, 40] were considered as high risk due to inappropriate allocation methods: one allocated participants based on age, and the other allocated participants based on their knowledge of the assigned group. (3) Blinding of researchers and subjects: Three studies [34, 39, 40] were considered to be at low risk and implemented blinding for participants and staff, while six studies [31, 35, 37, 42, 43] lacked detailed descriptions, and the risk of bias was unclear. Five studies [30, 32, 33, 38, 40] were considered to be at high risk and did not implement blinding for the staff. (4) Blinding evaluation of research results: Eight studies [30, 31, 34, 36, 38, 41] were considered to be at low risk and implemented blinding for evaluators. Four studies [35, 38, 42, 43] lacked detailed descriptions, and the risk of bias was unclear. Two studies [32, 33] were considered to be at high risk and did not implement blinding for evaluators. (5) Integrity of outcome data: Thirteen studies were considered to be at low risk. Specifically, 7 studies [33, 35–38, 41–43] demonstrated no loss to follow-up, while the remaining 6 studies [30, 32, 34, 38–40] reported loss to follow-up, with explanations provided for the reasons for the loss. One study [31] was considered to be at high risk and lost to follow-up without specifying the reason for the loss. (6) Selective reporting of research results: Two studies [30, 41] were registered in clinical trials with NCT numbers. The risk of bias in the remaining 12 studies was unknown, and the information provided for judgment was insufficient. (7) Other sources of bias: Only one study [39] was included. Due to the small sample size, only 14 patients were included and 5 were eventually lost to follow-up, which may pose a risk of bias. No other risk of bias was found in the rest of the articles. Details are shown in Figs. 2 and 3.

### Meta-analysis results of outcome measures

#### Comparison of changes in VAS pain scores

##### ARS group vs. physical therapy group

Five studies [31, 34, 40–42] compared the therapeutic effects of ARS and physical therapy on TMJ pain, and explored the differences in VAS score changes between the ARS group and physical therapy group at two treatment stages.

 [1] Follow-up duration of 0–3 months.

**Fig. 4:**
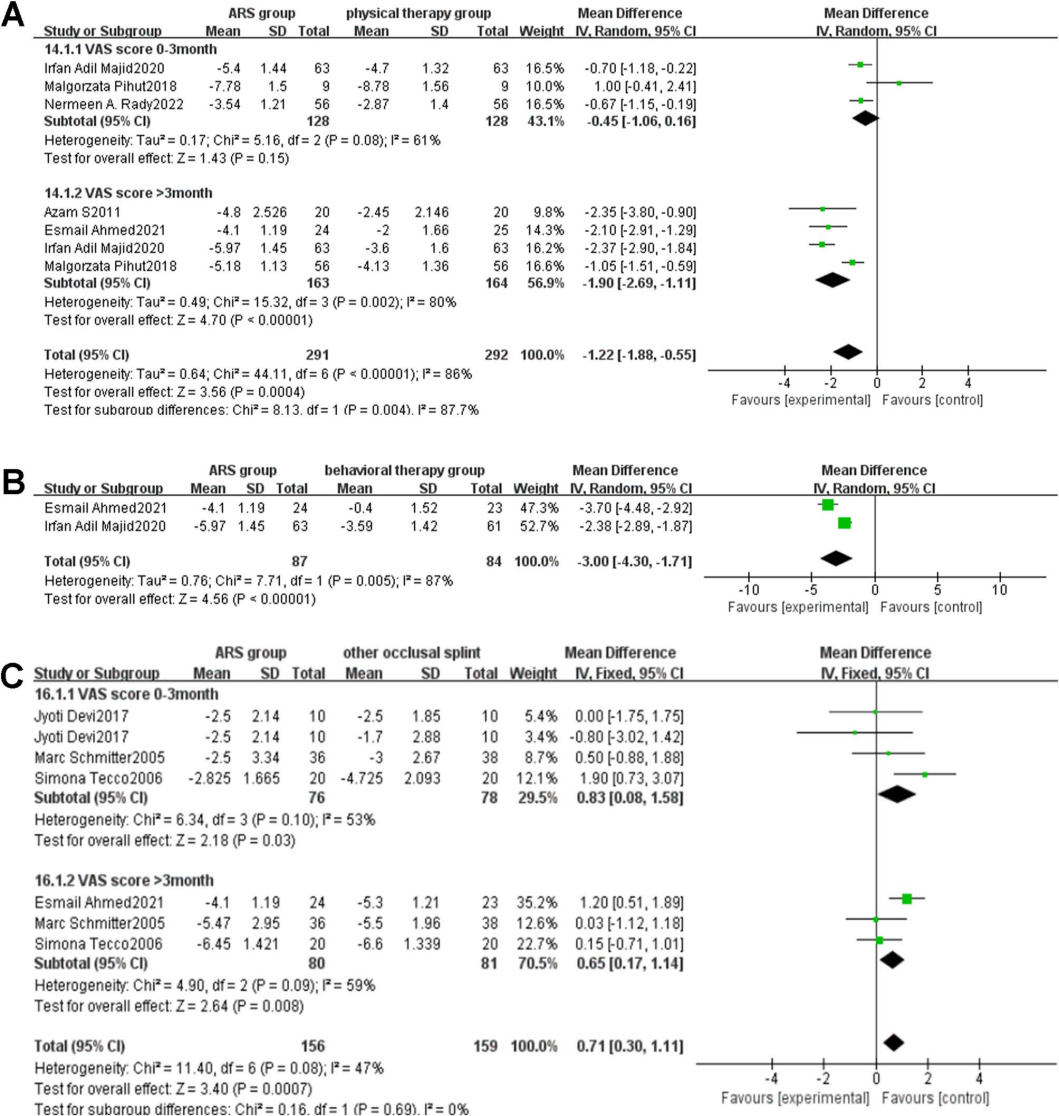
**A**: Forest plot of changes in VAS score (ARS group/physical therapy group) Note: A diamond marker falling to the left of the null line is more favorable for the ARS group; **B**: Forest plot of changes in VAS score (ARS group/behavioral therapy group) Note: A diamond marker falling to the left of the null line is more favorable for the ARS group; **C**: Forest plot of changes in VAS score (ARS group/other occlusal splint group) Note: A diamond marker falling to the right side of the null line is more favorable for other occlusal splint group

Three studies [40–42] reported the impact of VAS changes on patients in the ARS group and physical therapy group during 0–3 months of treatment. The analysis results are shown in the forest plot (Fig. 4A). The results of heterogeneity analysis indicated that there was significant heterogeneity among the articles (I²=61%, *P* = 0.08 < 0.10). According to statistical methods, a random-effects model was used to calculate pooled statistics of MD values. The analysis results showed that there was no statistically significant difference in VAS score improvement between the ARS group and the physical therapy group during 0–3 months of treatment [MD: -0.45, 95%CI (-1.06, 0.16), Z = 1.43, *P* = 0.15].

 [2] Follow-up duration of more than 3 months.

Four studies [31, 34, 40, 42] reported the impact of VAS changes on patients in the ARS group and physical therapy group after more than 3 months of treatment. The analysis results are shown in the forest plot (Fig. 4A). The results of heterogeneity analysis indicated that there was significant heterogeneity among the articles (I²=80%, *P* = 0.002 < 0.10). According to statistical methods, a random-effects model was used to calculate pooled statistics of MD values. In the forest plot, the combined effect size is located to the left of the null line, suggesting that the incidence rate in the intervention group (ARS group) is lower than that in the control group (physical therapy group). Since the VAS score, which is the event of interest in this study, is classified as an adverse event, a diamond marker falling to the left of the null line is more favorable for the intervention group (ARS group). The analysis results showed that there was a statistical difference in the improvement of VAS scores between the ARS group and the physical therapy group after more than 3 months of treatment with the ARS group showing significantly greater improvement than the physical therapy group [MD: -1.90, 95%CI (-2.69, -1.11), Z = 4.70, *P* < 0.00001].

##### ARS group vs. behavioral therapy group

Two studies [34, 40] reported changes in pain VAS scores in patients with TMJ disc displacement in the ARS group and behavioral therapy group, with a treatment duration exceeding three months. The analysis results are shown in the forest plot (Fig. 4B). The results of heterogeneity analysis indicated that there was significant heterogeneity among the articles (I²=87%, *P* = 0.005 < 0.10). According to statistical methods, a random-effects model was used to calculate pooled statistics of MD values. In the forest plot, the combined effect size is located to the left of the null line, suggesting that the incidence rate in the intervention group (ARS group) is lower than that in the control group (behavioral therapy group). Since the VAS score, which is the event of interest in this study, is classified as an adverse event, a diamond marker falling to the left of the null line is more favorable for the intervention group (ARS group). The analysis results showed that there was a statistical difference in the improvement of VAS scores between the ARS group and the behavioral therapy group after more than 3 months of treatment with the ARS group showing significantly greater improvement than the behavioral therapy group [MD=-3.00, 95%CI (-4.30, -1.71), *P* < 0.00001].

##### ARS group vs. other occlusal splint therapy group

Four studies [33, 34, 37, 43] compared the therapeutic effects of ARS with other occlusal splints on pain. Subgroup analysis based on treatment duration showed I²=47%. Therefore, a fixed-effects model was used for meta-analysis, as shown in the forest plot (Fig. 4C) [MD: 0.71, 95%CI (0.30, 1.11), Z = 3.4, *P* = 0.0007, I²=47%]. In the forest plot, the combined effect size is located to the right of the null line, suggesting that the incidence rate in the intervention group (ARS group) is higher than that in the control group (other occlusal splint group). Since the VAS score, which is the event of interest in this study, is classified as an adverse event, a diamond marker falling to the right of the null line is more favorable for the control group (other occlusal splint group). Both ARS and other occlusal splints could alleviate TMJ pain, and the results showed that whether in less than 3 months [MD: 0.83, 95%CI (0.08, 1.58), Z = 2.18, *P* = 0.03, I²=53%] or more than 3 months [MD: 0.65, 95%CI (0.17, 1.14), Z = 2.64, *P* = 0.008, I²=59%] of treatment, ARS was not as effective in relieving pain as other occlusal splints, and the difference was statistically significant.

#### Comparison of improvement in pain free mouth opening

##### ARS group vs. physical therapy group

The differences in the improvement of pain free mouth opening between the ARS group and the physical therapy group were compared at two treatment stages.

 [1] Follow-up duration of 0–3 months.

**Fig. 5:**
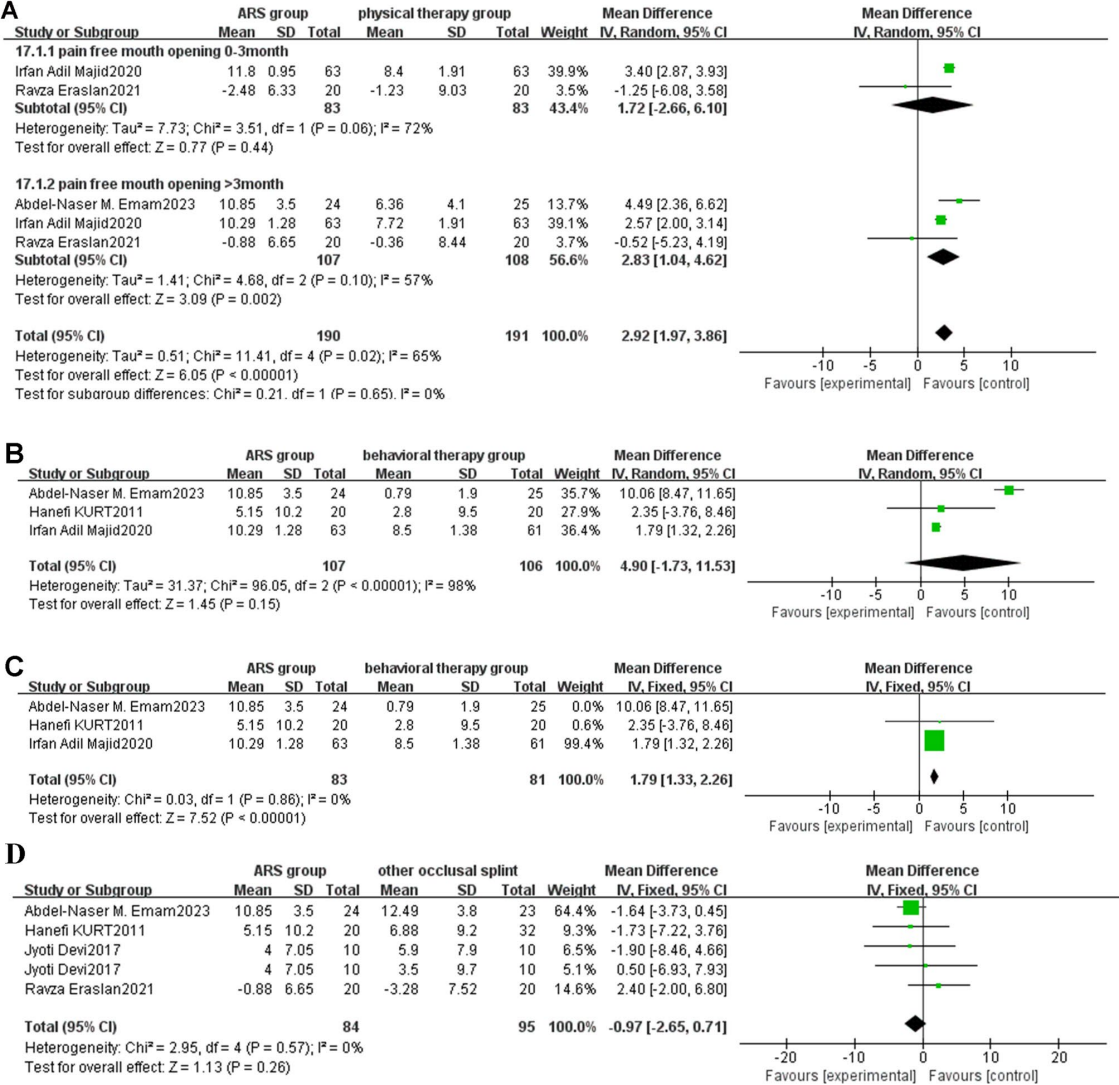
**A**: Forest plot of pain free mouth opening (ARS group/physical therapy group) Note: A diamond marker falling to the right side of the null line is more favorable for the ARS group; **B**: Forest plot of pain free mouth opening (ARS group/behavioral therapy group); **C**: Forest plot of pain free mouth opening (ARS group/behavioral therapy group) Note: A diamond marker falling to the right side of the null line is more favorable for the ARS group; **D** Forest plot of pain free mouth opening (ARS group/ other occlusal splint group)

Two studies [35, 40] measured and compared the pain free mouth opening of patients in the ARS group and physical therapy group during 0–3 months of treatment. The analysis results are shown in the forest plot (Fig. 5A). The results of heterogeneity analysis indicated that there was significant heterogeneity among the articles (I²=72%, *P* = 0.06 < 0.10). According to statistical methods, a random-effects model was used to calculate pooled statistics of MD values. The analysis results showed that there was no statistically significant difference in pain free mouth opening between the ARS group and the physical therapy group during 0–3 months of follow-up [MD = 1.72, 95%CI (-2.66, 6.10), *P* = 0.44].

 [2] Follow-up duration of more than 3 months.

Three studies [35, 36, 40] reported the impact of pain free mouth opening in the ARS group and physical therapy group after more than 3 months of treatment. The analysis results are shown in the forest plot (Fig. 5A). The results of heterogeneity analysis indicated that there was significant heterogeneity among the articles (I²=58%, *P* = 0.009 < 0.10). According to statistical methods, a random-effects model was used to calculate pooled statistics of MD values. In the forest plot, the combined effect size is located to the right of the null line, suggesting that the incidence rate in the intervention group (ARS group) is higher than that in the control group (physical therapy group). Since the VAS score, which is the event of interest in this study, is classified as an adverse event, a diamond marker falling to the right of the null line is more favorable for the intervention group (ARS group). The analysis results showed that there was a statistical difference between the ARS group and the physical therapy group in pain free mouth opening after more than 3 months of treatment, with the ARS group showing significantly greater improvement than the physical therapy group [MD = 2.83, 95%CI (1.04, 4.62), *P* = 0.002].

##### ARS group vs. behavioral therapy group

Three studies [32, 36, 40]compared the therapeutic effects of ARS and behavioral therapy on pain free mouth opening in TMD patients. The analysis results showed I²=98%. Therefore, a random-effects model was used for meta-analysis, as shown in the forest plot (Fig. 5B) [MD: 4.90, 95%CI (-1.73, 11.53), Z = 1.45, *P* < 0.00001, I²=98%]. There was no significant difference between ARS and behavioral therapy in improving pain free mouth opening, but ARS showed a trend of improving pain free mouth opening in TMD patients.

In the evaluation of the stability of the therapeutic effect of ARS and behavioral therapy on pain free mouth opening in TMD patients, the heterogeneity significantly decreased from 98 to 0% after removing Abdel Naser M. Emam’s study in 2023 [36], indicating that the excluded studies were the main source of significant heterogeneity in the results. The results are shown in Fig. 5C [MD: 1.79, 95%CI (1.33, 2.26), Z = 7.52, *P* < 0.00001, I²=0%]. In the forest plot, the combined effect size is located to the right of the null line, suggesting that the incidence rate in the intervention group (ARS group) is higher than that in the control group (behavioral therapy group). Since the pain free mouth opening, which is the event of interest in this study, is classified as a favourable event, a diamond marker falling to the right of the null line is more favorable for the intervention group (ARS group). The research findings indicated that ARS had a better effect on improving the pain free mouth opening of TMD patients than behavioral therapy, and the difference was statistically significant.

##### Comparison of the ARS group with other occlusal splint groups

Four studies [32, 35, 36, 43] compared the therapeutic effects of ARS versus other occlusal splints on pain free mouth opening in patients with TMD. The I² statistic was found to be 0%, leading to the application of a fixed-effects model for the meta-analysis. The results, presented in the forest plot (Fig. 5D), showed that there was no statistically significant difference between the ARS group and other occlusal splint groups in terms of improving pain free mouth opening in patients with TMD [MD: -0.97, 95%CI (-2.65, 0.71), Z = 1.13, (*P* = 0.26)].

#### Comparison of improvement in maximum active mouth opening

##### ARS group vs. physical therapy group

By measuring the maximum active mouth opening of patients at two treatment stages using different treatment methods, a comparative study was conducted to compare the differences between the ARS group and the physical therapy group.

 [1] Follow-up duration of 0–3 months.

**Fig. 6:**
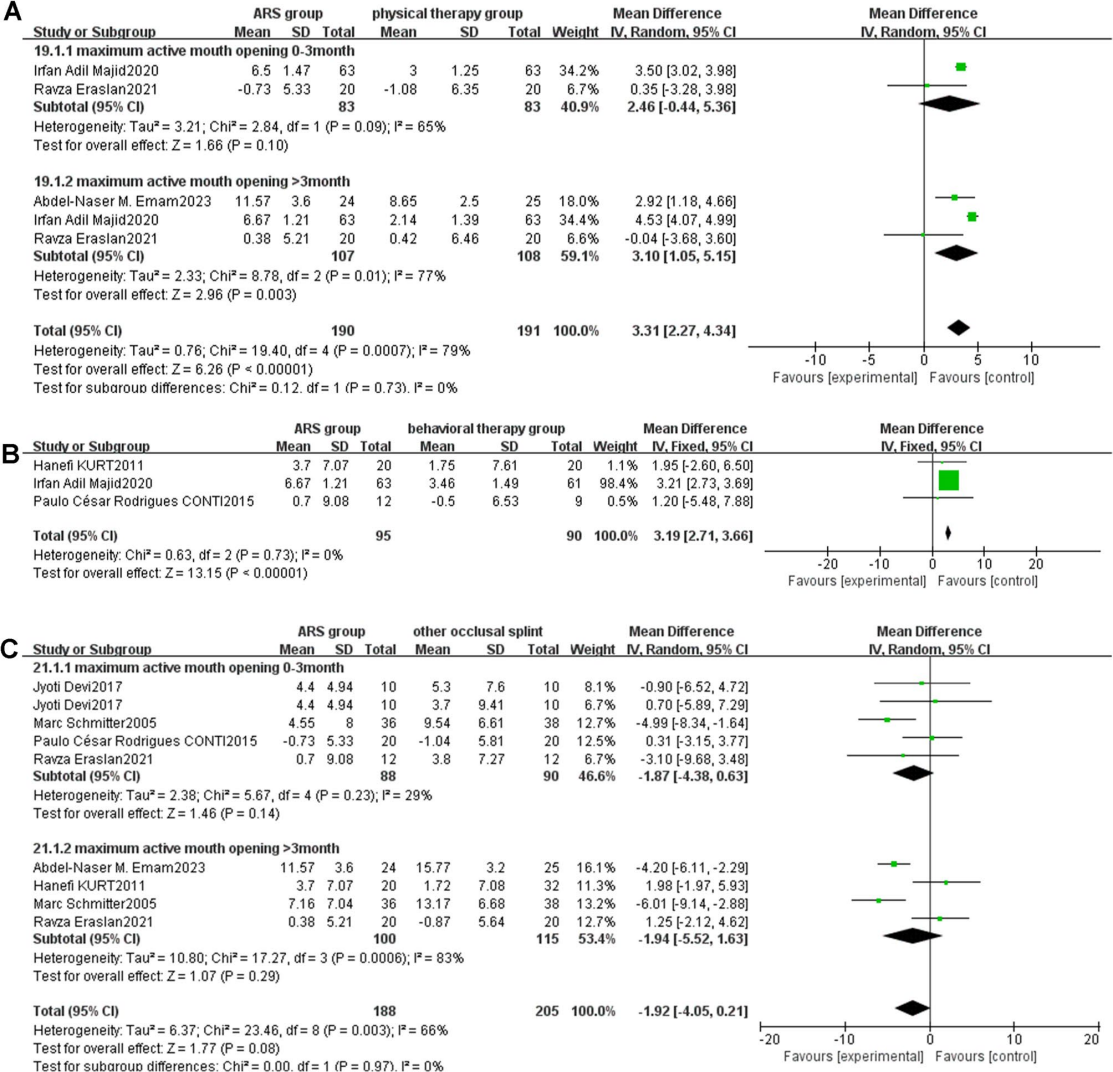
**A**: Forest plot of maximum active mouth opening (ARS group/physical therapy group) Note: A diamond marker falling to the right side of the null line is more favorable for the ARS group; **B**: Forest plot of maximum active mouth opening (ARS group/behavioral therapy group) Note: A diamond marker falling to the right side of the null line is more favorable for the ARS group; **C**: Forest plot of maximum active mouth opening (ARS group/other occlusal splint group)

Two studies [35, 40]measured the maximum active mouth opening of patients in the ARS group and physical therapy group during 0–3 months of treatment, and compared using appropriate methods. The pooled analysis results are shown in the forest plot (Fig. 6A). The results of heterogeneity analysis indicated I²=65% (*P* = 0.09 < 0.10). A random-effects model was used to calculate pooled statistics of MD values. The analysis results [MD: 2.46, 95%CI (-0.44,5.36), Z = 1.66, *P* = 0.10] showed that there was no statistically significant difference in maximum active mouth opening between the ARS group and the physical therapy group during 0–3 months of treatment (*P* = 0.10).

 [2] Follow-up duration of more than 3 months.

Three studies [35, 36, 40] reported the impact on maximum active mouth opening in the ARS group and physical therapy group after more than 3 months of treatment. The analysis results are shown in the forest plot (Fig. 6A). The results of heterogeneity analysis indicated I²=77% (*P* = 0.01 < 0.10). A random-effects model was used to calculate pooled statistics of MD values. In the forest plot, the combined effect size is located to the right of the null line, suggesting that the incidence rate in the intervention group (ARS group) is higher than that in the control group (physical therapy group). Since the maximum active mouth opening, which is the event of interest in this study, is classified as a favourable event, a diamond marker falling to the right of the null line is more favorable for the intervention group (ARS group). The analysis results showed that there was a statistical difference in the improvement of maximum active mouth opening between the ARS group and the physical therapy group after more than 3 months of treatment, with the ARS group showing significantly greater improvement than the physical therapy group [MD: 3.10, 95%CI (1.05, 5.15), *P* = 0.003].

##### ARS group vs. behavioral therapy group

Three studies [32, 38, 40] compared the therapeutic effects of ARS and behavioral therapy on maximum active mouth opening in TMD patients. The analysis showed I²=0%. Therefore, a fixed-effects model was used for meta-analysis, as shown in the forest plot (Fig. 6B) [MD: 3.19, 95%CI (2.71, 3.66), Z = 13.15, *P* < 0.00001, I²=0%]. In the forest plot, the combined effect size is located to the right of the null line, suggesting that the incidence rate in the intervention group (ARS group) is higher than that in the control group (behavioral therapy group). Since the maximum active mouth opening, which is the event of interest in this study, is classified as a favourable event, a diamond marker falling to the right of the null line is more favorable for the intervention group (ARS group). Both ARS and behavioral therapy could improve maximum active mouth opening. However, the ARS group showed a better effect, and the difference was statistically significant.

##### ARS group vs. other occlusal splint therapy group

Six studies [32, 33, 35, 36, 38, 43] compared the therapeutic effects of ARS and other occlusal splints on maximum active mouth opening in TMD patients. Subgroup analysis based on treatment duration showed I²=66%. Therefore, a random-effects model was used for meta-analysis, as shown in the forest plot (Fig. 6C) [MD: -1.92, 95%CI (-4.05, 0.21), Z = 1.77, *P* = 0.08, I²=66%]. In the comparison between ARS and other occlusal splints, whether in less than 3 months [MD: -1.84, 95%CI (-4.38, 0.63), Z = 1.46, *P* = 0.14, I²=29%] or more than 3 months [MD: -1.94, 95%CI (-5.52, 1.63), Z = 1.07, *P* = 0.29, I²=83%] of treatment, both treatments could improve maximum active mouth opening, and there was no significant difference between the two groups.

#### Comparison of improvement in maximum passive mouth opening

##### ARS group vs. physical therapy group

**Fig. 7:**
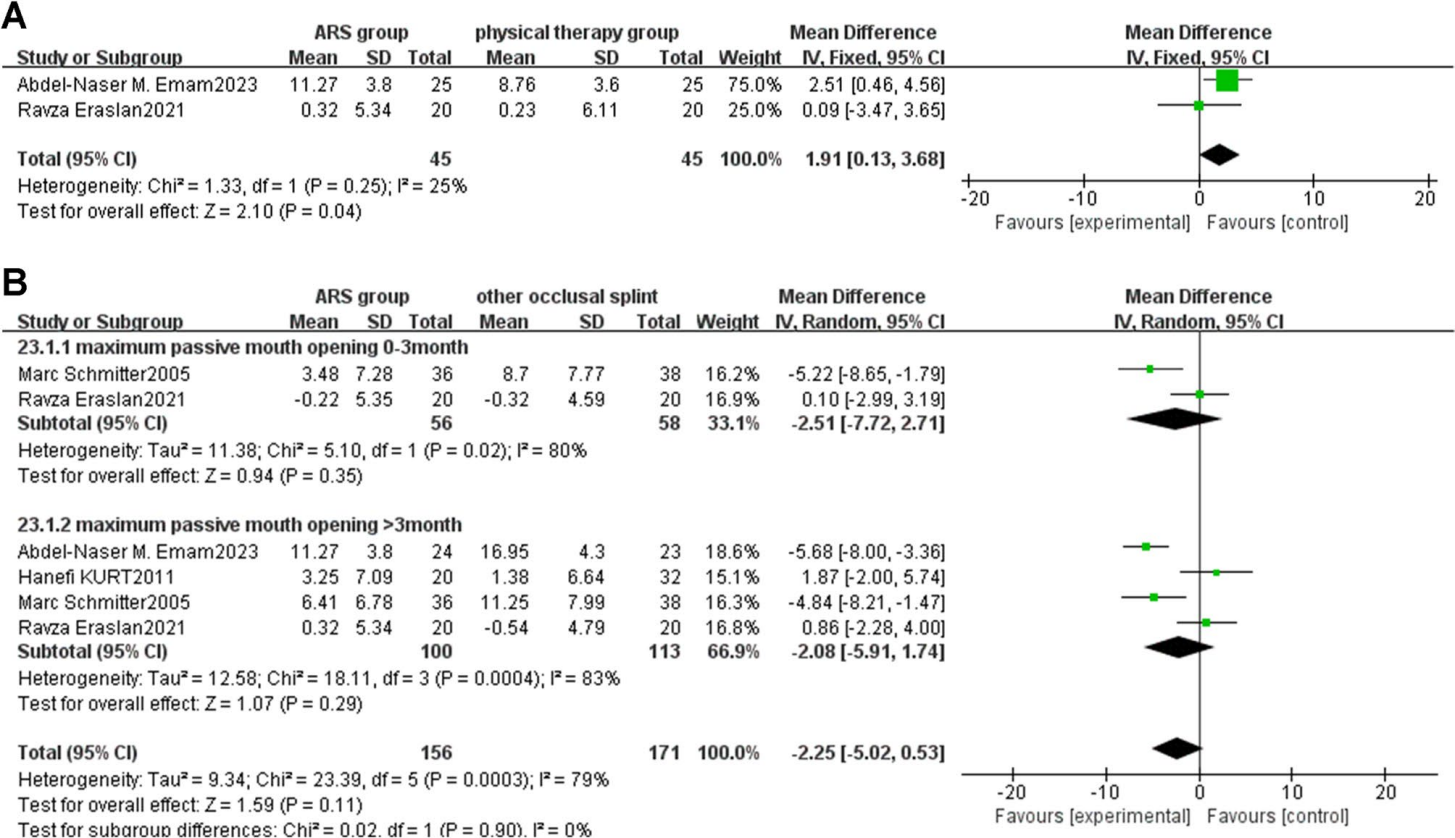
**A** Forest plot of maximum passive mouth opening (ARS group/physical therapy group) Note: A diamond marker falling to the right side of the null line is more favorable for the ARS group; **B**: Forest plot of maximum passive mouth opening (ARS group/other occlusal splint therapy group)

Two studies [35, 36] compared the therapeutic effects of ARS and physical therapy on maximum passive mouth opening in TMD patients. The analysis showed I²=25%. Therefore, a fixed-effects model was used for meta-analysis, as shown in the forest plot (Fig. 7A) [MD: 1.91, 95%CI (0.13, 3.68), Z = 2.10, *P* = 0.04, I²=25%]. In the forest plot, the combined effect size is located to the right of the null line, suggesting that the incidence rate in the intervention group (ARS group) is higher than that in the control group (physical therapy group). Since the maximum passive mouth opening, which is the event of interest in this study, is classified as a favourable event, a diamond marker falling to the right of the null line is more favorable for the intervention group (ARS group). In the comparison between ARS and physical therapy, both treatments could improve maximum passive mouth opening. However, the improvement degree of the ARS group was greater than that of the physical therapy group, and the difference was statistically significant.

##### ARS group vs. other occlusal splint therapy group

Four studies [32, 33, 35, 36] compared the therapeutic effects of ARS and other occlusal splints on maximum passive mouth opening in TMD patients. Subgroup analysis based on treatment duration showed I²=79%. Therefore, a random-effects model was used for meta-analysis, as shown in the forest plot (Fig. 7B) [MD: -2.25, 95%CI (-5.02, 0.53), Z = 1.59, *P* = 0.11, I²=79%]. In the comparison between ARS and other occlusal splints, whether in less than 3 months [MD: -2.51, 95%CI (-7.72, 2.71), Z = 0.94, *P* = 0.35, I²=80%] or more than 3 months [MD: -2.08, 95%CI (-5.91, 1.74), Z = 1.07, *P* = 0.29, I²=83%] of treatment, both treatments could improve maximum passive mouth opening, and there was no significant difference between the two groups.

#### Comparison of alleviation in popping

##### ARS group vs. physical therapy group

**Fig. 8:**
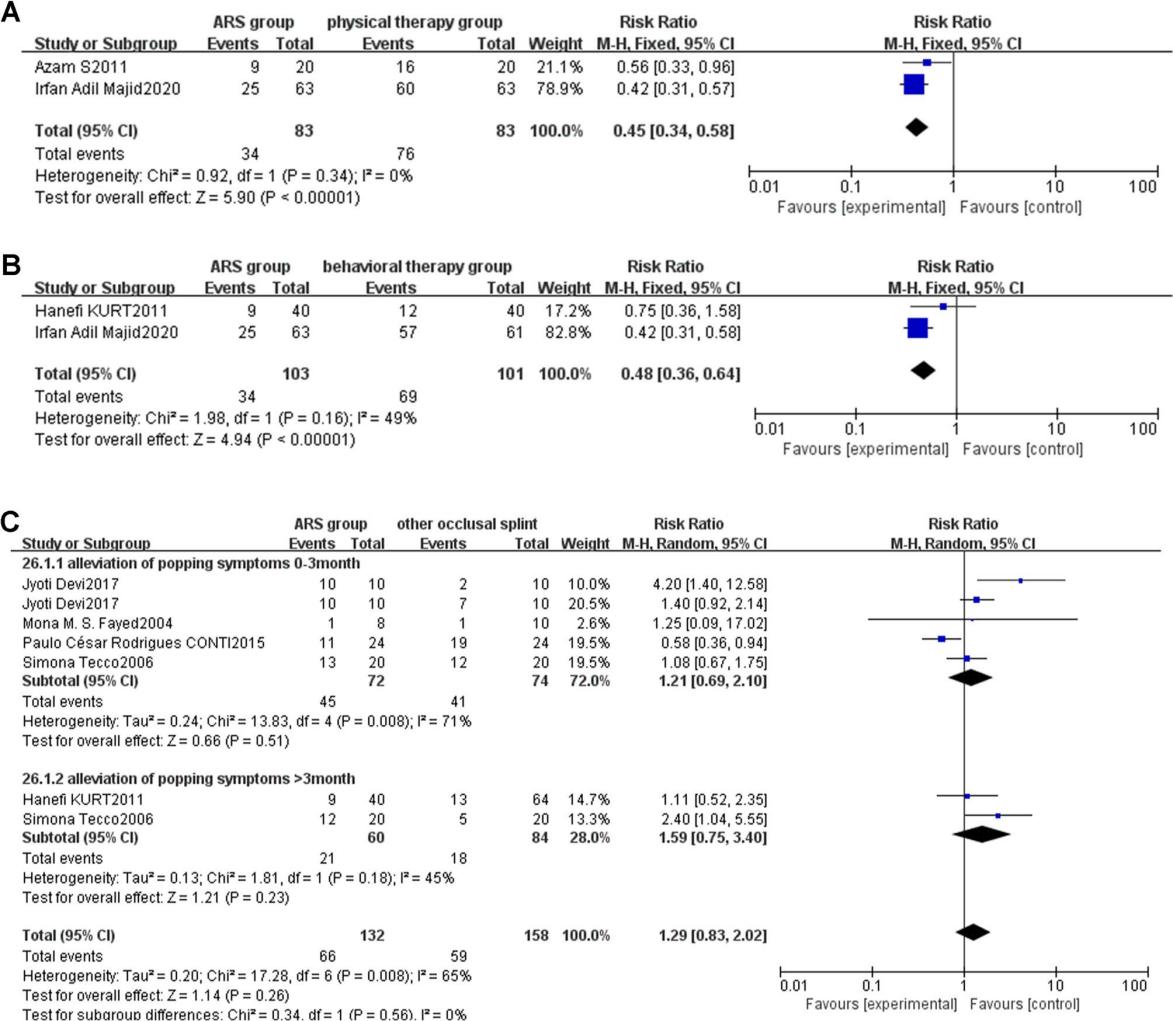
**A**: Forest plot of alleviation of popping symptoms (ARS group/physical therapy group) Note: A diamond marker falling to the left of the null line is more favorable for the ARS group; **B**: Forest plot of alleviation of popping symptoms (ARS group/behavioral therapy group) Note: A diamond marker falling to the left of the null line is more favorable for the ARS group; **C**: Forest plot of alleviation of popping symptoms (ARS group/other occlusal splint therapy group)

Two studies [31, 40] compared the therapeutic effects of ARS and physical therapy on TMJ popping. Subgroup analysis showed I²=0%. Therefore, a fixed-effects model was used for meta-analysis, as shown in the forest plot (Fig. 8A) [RR: 0.45, 95%CI (0.34, 0.58), Z = 5.90, *P* < 0.00001, I²=0%]. In the forest plot, the combined effect size is located to the left of the null line, suggesting that the incidence rate in the intervention group (ARS group) is lower than that in the control group (physical therapy group). Since the popping, which is the event of interest in this study, is classified as an adverse event, a diamond marker falling to the left of the null line is more favorable for the intervention group (ARS group). In the comparison between ARS and physical therapy, both treatments could alleviate TMJ popping. However, ARS showed a better effect, and the difference was statistically significant.

##### ARS group vs. behavioral therapy group

Two studies [32, 40] compared the therapeutic effects of ARS and behavioral therapy on TMJ popping. Subgroup analysis showed I²=49%. Therefore, a fixed-effects model was used for meta-analysis, as shown in the forest plot (Fig. 8B) [RR: 0.48, 95%CI (0.36, 0.64), Z = 4.94, *P* = 0.00001]. In the forest plot, the combined effect size is located to the left of the null line, suggesting that the incidence rate in the intervention group (ARS group) is lower than that in the control group (behavioral therapy group). Since the popping, which is the event of interest in this study, is classified as an adverse event, a diamond marker falling to the left of the null line is more favorable for the intervention group (ARS group). In the comparison between ARS and behavioral therapy, both treatments could alleviate TMJ popping. However. ARS showed a better effect, and the difference was statistically significant.

##### ARS group vs. other occlusal splint therapy group

Four studies [32, 37, 39, 43] compared the therapeutic effects of ARS and other occlusal splints on TMJ popping. Subgroup analysis based on treatment duration showed I²=65%. Therefore, a random-effects model was used for meta-analysis, as shown in the forest plot (Fig. 8C) [RR: 1.29, 95%CI (0.83, 2.02), *P* = 0.26]. The results showed that in the comparative study of ARS and other occlusal splints, both could effectively alleviate TMJ popping, regardless of the treatment duration being less than 3 months [RR: 1.21, 95%CI (0.69, 2.10), *P* = 0.51] or greater than 3 months [RR: 1.59, 95%CI (0.75, 3.40), *P* = 0.23], and the difference was not statistically significant.

### Sensitivity analysis and publication bias assessment

By sequentially excluding each study and observing the pooled results of the remaining studies, the results remained largely consistent with the overall result, indicating the stability and reliability of the included studies. Detailed information can be found in the supplementary files(Figure S1-S8).

Publication bias was assessed using funnel plots for the outcomes of maximum active mouth opening, pain free mouth opening, and VAS score in the comparison of the ARS group versus the physical therapy group, and for the outcomes of maximum active mouth opening, maximum passive mouth opening, pain free mouth opening, popping, and VAS score in the comparison of the ARS group versus other occlusal splint treatment groups. Evidence of asymmetry was observed in the results, raising concerns about the possibility of publication bias. Further details are available in the supplementary files(Figure S9-S16).

### GRADE evidence assessment

The quality of evidence for each outcome measure included in the meta-analysis was assessed using the GRADE approach. The assessment revealed that the evidence quality for maximum passive mouth opening (ARS group vs. physical therapy group) and popping (ARS group vs. physical therapy group) was moderate. The evidence quality for VAS (ARS group vs. physical therapy group) > 3 months, pain free mouth opening (ARS group vs. behavioral therapy group), maximum active mouth opening(ARS group vs. other occlusal splint therapy group), maximum passive mouth opening (ARS group vs. other occlusal splint therapy group) > 3 months, popping (ARS group vs. behavioral therapy group), and popping (ARS group vs. other occlusal splint therapy group) > 3 months was low. The remaining 12 outcome measures were rated as very low quality (Table 3).

**Table 3:** Summary of findings

Certainty assessment							№ of patients		Effect		Certainty	Importance
№ of studies	Study design	Risk of bias	Inconsistency	Indirectness	Imprecision	Other considerations	Intervention	comparison	Relative (95% CI)	Absolute (95% CI)		
**VAS( ARS group vs. physical therapy group)0-3month**												
3	randomised trials	not serious	serious^a^	not serious	serious^b^	publication bias strongly suspected^c^	128	128	-	MD **0.45 lower** (1.06 lower to 0.16 higher)	⨁◯◯◯ Very low^a, b,c^	CRITICAL
**VAS( ARS group vs. physical therapy group) > 3month**												
4	randomised trials	not serious	serious^a^	not serious	not serious	publication bias strongly suspected^c^	163	164	-	MD **1.9 lower** (2.69 lower to 1.11 lower)	⨁⨁◯◯ Low^a, c^	CRITICAL
**VAS( ARS group vs. behavioral therapy group)**												
2	randomised trials	serious^d^	serious^a^	not serious	not serious	publication bias strongly suspected^c^	87	84	-	MD **3 lower** (4.3 lower to 1.71 lower)	⨁◯◯◯ Very low^a, c,d^	CRITICAL
**VAS(ARS group vs. other occlusal splint therapy group)0-3month**												
4	randomised trials	serious^d^	serious^a^	not serious	serious^b^	publication bias strongly suspected^c^	76	78	-	MD **0.83 higher** (0.08 higher to 1.58 higher)	⨁◯◯◯ Very low^a, b,c, d^	CRITICAL
**VAS(ARS group vs. other occlusal splint therapy group) > 3month**												
3	randomised trials	serious^d^	serious^a^	not serious	not serious	publication bias strongly suspected^c^	80	81	-	MD **0.65 higher** (0.17 higher to 1.14 higher)	⨁◯◯◯ Very low^a, c,d^	CRITICAL
**pain free mouth opening(ARS group vs. physical therapy group)0-3month**												
2	randomised trials	serious^d^	serious^a^	not serious	serious^e^	publication bias strongly suspected^c^	83	83	-	MD **1.72 higher** (2.66 lower to 6.1 higher)	⨁◯◯◯ Very low^a, c,d, e^	CRITICAL
**pain free mouth opening(ARS group vs. physical therapy group) > 3month**												
3	randomised trials	serious^d^	serious^a^	not serious	not serious	publication bias strongly suspected^c^	107	108	-	MD **2.83 higher** (1.04 higher to 4.62 higher)	⨁◯◯◯ Very low^a, c,d^	CRITICAL
**pain free mouth opening(ARS group vs. behavioral therapy group)**												
2	randomised trials	serious^d^	not serious	not serious	not serious	publication bias strongly suspected^c^	83	81	-	MD **1.79 higher** (1.33 higher to 2.26 higher)	⨁⨁◯◯ Low^c, d^	CRITICAL
**pain free mouth opening(ARS group vs. other occlusal splint therapy group)**												
5	randomised trials	serious^d^	not serious	not serious	serious^b^	publication bias strongly suspected^c^	84	95	-	MD **0.97 lower** (2.65 lower to 0.71 higher)	⨁◯◯◯ Very low^b, c,d^	CRITICAL
**maximum active mouth opening( ARS group vs. physical therapy group)0-3month**												
2	randomised trials	serious^d^	serious^a^	not serious	serious^b, e^	publication bias strongly suspected^c^	83	83	-	MD **2.46 higher** (0.44 lower to 5.36 higher)	⨁◯◯◯ Very low^a, b,c, d,e^	CRITICAL
**maximum active mouth opening( ARS group vs. physical therapy group) > 3month**												
3	randomised trials	serious^d^	serious^a^	not serious	serious^b^	publication bias strongly suspected^c^	107	108	-	MD **3.1 higher** (1.05 higher to 5.15 higher)	⨁◯◯◯ Very low^a, b,c, d^	CRITICAL
**maximum active mouth opening(ARS group vs. behavioral therapy group)**												
3	randomised trials	serious^d^	not serious	not serious	serious^b^	publication bias strongly suspected^c^	95	90	-	MD **3.19 higher** (2.71 higher to 3.66 higher)	⨁◯◯◯ Very low^b, c,d^	CRITICAL
**maximum active mouth opening(ARS group vs. other occlusal splint therapy group)0-3month**												
5	randomised trials	serious^d^	not serious	not serious	serious^b^	none	88	90	-	MD **1.87 lower** (4.38 lower to 0.63 higher)	⨁⨁◯◯ Low^b, d^	CRITICAL
**maximum active mouth opening(ARS group vs. other occlusal splint therapy group) > 3month**												
4	randomised trials	serious^d^	serious^a^	not serious	not serious	none	100	115	-	MD **1.94 lower** (5.52 lower to 1.63 higher)	⨁⨁◯◯ Low^a, d^	CRITICAL
**maximum passive mouth opening(ARS group vs. physical therapy group)**												
2	randomised trials	not serious	not serious	not serious	not serious	publication bias strongly suspected^c^	45	45	-	MD **1.91 higher** (0.13 higher to 3.68 higher)	⨁⨁⨁◯ Moderate^c^	CRITICAL
**maximum passive mouth opening( ARS group vs. other occlusal splint therapy group)0-3month**												
2	randomised trials	serious^d^	serious^a^	not serious	not serious	publication bias strongly suspected^e^	56	58	-	MD **2.51 lower** (7.72 lower to 2.71 higher)	⨁◯◯◯ Very low^a, d,e^	CRITICAL
**maximum passive mouth opening( ARS group vs. other occlusal splint therapy group) > 3month**												
4	randomised trials	serious^d^	serious^a^	not serious	not serious	none	100	113	-	MD **2.25 lower** (5.91 lower to 1.74 higher)	⨁⨁◯◯ Low^a, d^	CRITICAL
**popping( ARS group vs. physical therapy group)**												
2	randomised trials	serious^d^	not serious	not serious	not serious	none	34/83 (41.0%)	76/83 (91.6%)	**RR 0.45** (0.34 to 0.58)	**504 fewer per 1**,**000** (from 604 fewer to 385 fewer)	⨁⨁⨁◯ Moderate^d^	CRITICAL
**popping(ARS group vs. behavioral therapy group)**												
2	randomised trials	serious^d^	not serious	not serious	not serious	publication bias strongly suspected^c^	34/103 (33.0%)	69/101 (68.3%)	**RR 0.48** (0.36 to 0.64)	**355 fewer per 1**,**000** (from 437 fewer to 246 fewer)	⨁⨁◯◯ Low^c, d^	CRITICAL
**popping( ARS group vs. other occlusal splint therapy group)0-3month**												
5	randomised trials	serious^d^	serious^a^	not serious	serious^b^	publication bias strongly suspected^c^	45/72 (62.5%)	41/74 (55.4%)	**RR 1.21** (0.69 to 2.10)	**116 more per 1**,**000** (from 172 fewer to 609 more)	⨁◯◯◯ Very low^a, b,c, d^	CRITICAL
**popping( ARS group vs. other occlusal splint therapy group) > 3month**												
2	randomised trials	serious^d^	not serious	not serious	not serious	publication bias strongly suspected^c^	21/60 (35.0%)	18/84 (21.4%)	**RR 1.59** (0.75 to 3.40)	**13 more per 100** (from 5 fewer to 51 more)	⨁⨁◯◯ Low^c, d^	CRITICAL

**CI**: confidence interval; **MD**: mean difference; **RR**: risk ratio

**Explanations**

a. I^2^ is greater than 50% and there is heterogeneity

b. The included studies had small sample sizes

c. Funnel charts show asymmetry or no funnel plots

d. One study had high overall risk of bias

e. The confidence interval of the aggregated results is wide

## Analysis and discussion

The conservative treatment methods for TMJ disc displacement are varied and numerous. Nevertheless, there is no consensus on the most effective treatment method to date. The specific conditions of patients, including the type of disc displacement, symptoms, disease course, and age, all influence the choice of treatment. By comparing the efficacy of different treatment methods, more effective treatment plans can be found to improve the treatment outcomes and quality of life for patients. The present systematic review and meta-analysis aimed to assess the clinical effectiveness of repositioning occlusal splints in the management of disc displacement, in comparison to alternative conservative modalities. Other conservative treatments included in this research were: stabilization occlusal splints, canine guidance occlusal splints, NTI-tss occlusal splints, SVED and MORA occlusal splints, soft resilient occlusal splints, low-level laser therapy (LLLT), behavioral therapy, and physical therapy. Through comparative effectiveness research, this study objectively evaluated the therapeutic outcomes of various treatment modalities for TMJ disc displacement, with the aim of providing evidence for standardized management of the condition.

### Evidence quality grading results analysis

In this study, the GRADE evidence grading classified 2 outcome measures as moderate quality, 7 as low-quality evidence, and 12 as very low-quality evidence. These findings may affect the reliability of the conclusions drawn from this research. Furthermore, the risk of bias for most outcome measures was downgraded by one level. The decreased confidence in the evidence was primarily due to inadequate randomization and blinding, inconsistency of results, and reporting bias. Consequently, future research should prioritize standardized experimental designs and rigorous study execution.

### Comparison of ARS treatment and physical therapy

Compared to physical therapy, ARS showed no significant differences in pain VAS scores, pain free mouth opening, and maximum active mouth opening during the 0–3 month follow-up period. Following a follow-up period of more than 3 months, statistically significant differences were observed between the two groups in VAS pain scores, pain free mouth opening, maximum active mouth opening, maximum passive mouth opening, and alleviation in popping symptoms. This indicated that ARS demonstrated superior efficacy in comparison to physical therapy. This finding was consistent with the conclusion of Cai Bin [44], who suggested that physical therapy was often more effective for anterior disc displacement without reduction (ADDWoR) patients with limited mouth opening and pain duration ≤ 2 months. In chronic ADDwoR patients, particularly elderly individuals with joint pain complicated by osteoarthritis, physical therapy alone is often insufficient. A combined approach incorporating medication and ARS therapy is typically required.

### Comparison of ARS treatment and behavioral therapy

Both ARS and behavioral therapy demonstrated efficacy in improving pain free mouth opening, VAS pain scores, maximum active mouth opening, and alleviating popping symptoms after a follow-up period of more than 3 months. However, ARS exhibited superior efficacy compared to behavioral therapy. This finding was consistent with the result from a previous RCT reported by Shedden Mora MC et al. [45]. They randomly assigned 58 patients with chronic TMD to either 8 weeks of behavioral therapy or 8 weeks of occlusal splint therapy. The result showed that both treatments effectively alleviated pain and improved mandibular motor function, with success rates of 45% and 48%, respectively. Behavioral therapy, including patient education, function training, and behavioral guidance, constitutes the foundation of TMD treatment. TMD is a complex disorder resulting from the interplay of genetic predisposition, environmental influences, psychological factors, and behavioral patterns. The biopsychosocial model is now widely accepted by researchers in the analysis and treatment of TMD [46, 47]. Some researchers [48] suggest that conservative treatment alone can alleviate symptoms such as pain and limited mouth opening in TMD patients. However, combining behavioral therapy can enhance treatment efficacy. Future studies may consider the use of ARS in conjunction with behavioral therapy for the treatment of disc displacement.

### Comparison of ARS treatment and other occlusal splint therapies

Compared to other occlusal splint therapies, ARS showed no significant statistical differences in the improvement of pain free mouth opening, maximum active mouth opening, maximum passive mouth opening, and the reduction of popping symptoms over the entire follow-up period. In terms of alleviating TMJ pain, ARS did not demonstrate superior efficacy compared to other types of occlusal splints. This finding aligned with a systematic review and meta-analysis by Maheshwari K et al. [26], which demonstrated no significant difference between ARS and other types of occlusal splints in the treatment of disc displacement with reduction (DDwR). Ferrillo M et al. [49] performed a meta-analysis on the efficacy of conservative treatments for TMJ pain, concluding that modified hard stabilization splints were the most effective for improving pain in patients with temporomandibular joint disorders, followed by soft resilient splints, repositioning splints, and traditional stabilization splints. This aligned with the findings of the present study. Devi J et al. [43] randomly assigned 30 patients diagnosed with DDwR into three groups, treating them with stabilization splints, ARS, and soft splints, respectively. Follow-up revealed that patients in the stabilization splint group showed more significant improvements in mouth opening, clicking, and pain reduction compared to the other groups. Therefore, it is recommended that patients in the acute phase of DDwR be treated with stabilization splints to achieve faster and more effective outcomes with minimal side effects.

Some previous research conclusions were inconsistent with this findings. Al-Moraissi et al. [20] conducted a meta-analysis on the effectiveness of various types of splints in treating TMD. Although the quality of evidence was low, the results showed that ARS showed better therapeutic effects in reducing pain and popping compared to other splints. This discrepancy may be attributed to the mechanism of action of ARS, which involves recapturing the displaced articular disc via mandibular advancement. This, in turn, restores normal disc-condyle relationship and the biomechanical properties of joint function, reduces intracapsular pressure, and consequently alleviates pain, popping, and improves mandibular motor function [50]. This systematic evaluation included 14 RCTs. Among these, 7 studies [30, 33, 34, 36, 37, 39, 41] confirmed the diagnosis using MRI. The remaining 7 studies [31, 32, 35, 38, 40, 42, 43] diagnosed patients based on the RDC/TMD diagnostic criteria through clinical examination and questionnaire surveys, without performing MRI. Therefore, the exact disc-condyle relationship could not be determined in these studies. Patients may present with anterior disc displacement concurrently with medial or lateral displacement, and the condyle may exhibit rotation or angulation. Due to these variations in disease characteristics, mandibular advancement may not fully alleviate the symptoms. Within the studies included in this systematic review, only two [39, 41] utilized MRI at the 3-month follow-up to assess treatment outcomes. The majority of studies relied on clinical symptoms such as popping, pain, and mandibular function as primary outcome measures, rather than structural improvements. The alleviation of clinical symptoms, including the reduction of muscle pain and joint pain, does not necessarily imply a full restoration of the disc-condyle relationship. The intra-articular pressure may have been improved, but anterior disc displacement still exits. Some studies have demonstrated successful recapture of the articular disc in the early stages of application. However, subsequent occlusal splint adjustment and gradual reduction have been observed to correlate with anterior redisplacement of the disc. Other occlusal splints, especially stabilization splint, can expand the temporomandibular joint space, change the vertical distance, and achieve good therapeutic effects in relieving TMJ pain by enhancing occlusal stability, reducing muscle tension, and forming functional balance in stomatognathic system [51]. This may explain why our study failed to demonstrate a significant advantage of ARS over other types of occlusal splints in terms of pain relief, maximal mouth opening, and popping alleviation.

ARS is commonly used for the treatment of DDwR. The mechanism of action involves moving the condyle anteriorly and inferiorly to recapture the anteriorly displaced disc and reduce intra-articular pressure, thereby treating disc displacement. In adolescents with DDwR, these splints also promote condylar growth and remodeling [52]. Compared to stabilization occlusal splints, ARS demonstrates a higher rate of disc recapture [53], but is also associated with a higher recurrence rate [15, 54]. Treatment success may be influenced by factors such as disease duration, the degree of disc displacement, and the patient’s occlusal relationship. To maintain long-term disc-condyle stability, orthodontic treatment or occlusal reconstruction may be necessary [55]. Stabilization occlusal splints are effective in increasing the joint space, thereby alleviating pain and improving mandibular function, particularly in patients with TMD characterized by arthralgia or DDWoR. However, they should be used in conjunction with other conservative treatments, such as physical therapy and joint lavage, to maximize therapeutic outcomes [13]. Clinical decisions regarding treatment strategies should be tailored to the individual patient’s condition (e.g., disc status, acute versus chronic symptoms) based on a comprehensive multidisciplinary assessment (including imaging and occlusal analysis).

### Limitations in this study

Due to the limitations in the level of systematic reviews and the quality of included studies, this systematic review and meta-analysis still have certain deficiencies. Firstly, the restriction to English-language publications may have resulted in selection bias, as relevant studies published in other languages were not considered. Secondly, this research did not perform subgroup analyses to evaluate the effect of occlusal splint wearing time on treatment outcomes. Lastly, the absence of MRI imaging during the follow-up period in most of the included studies restricts our evaluation to clinical symptoms, including popping, pain, and mandibular movement function. Consequently, we are unable to track and assess structural changes in the TMJ before and after treatment.

### Future perspectives

It is anticipated that future investigations will feature high-quality, multicenter, large-sample RCTs. These studies should integrate pre-treatment and post-treatment MRI examinations and the recording of mandibular condyle movement trajectories to monitor improvements in structures related to disc repositioning during follow-up periods. Additionally, we hope that future research will include comprehensive data on the use of ARS. This information will facilitate a more thorough analysis of how wear time influences treatment outcomes, thereby enhancing our understanding of the short-term and long-term efficacy of ARS in managing TMD.

## Conclusion

A total of 14 studies were included in this systematic review and meta-analysis, which compared the efficacy of ARS with other conservative treatments for disc displacement. While ARS did not demonstrate a significant difference compared to physical therapy in improving TMJ pain, popping, and mandibular motor function in the initial treatment phase, it exhibited more pronounced effects than physical therapy and behavioral therapy with continued use. Splints have demonstrated significant efficacy in alleviating TMJ popping and improving mandibular movement. However, ARS has not shown superior effectiveness compared to other types of occlusal splints in alleviating pain.

## Electronic supplementary material

Below is the link to the electronic supplementary material.

Supplementary Material 1

## Data Availability

The datasets generated during and/or analysed during the current study are available from the corresponding author on reasonable request.

## Abbreviations

ARS: Anterior Repositioning Splint
TMJ: Temporomandibular Joint
RCTs: Randomized Controlled Trials
TMD: Temporomandibular Disorders
PICOS: Participants, Interventions, Comparisons, Outcomes, and Studies
DC/TMD: Diagnostic Criteria for Temporomandibular Disorders
ID: Internal Derangement
ADDWR: Anterior Disc Displacement with Reduction
ADDWoR: Anterior Disc Displacement without Reduction
MRI: Magnetic Resonance Imaging
PRISMA: Preferred Reporting Items for Systematic Reviews and Meta-Analysis guidelines
RDC/TMD: Research Diagnostic Criteria for Temporomandibular Disorders
VAS: Visual Analogue Scale
DD: Disc Displacement
I²: Heterogeneity Index
WMD: Weighted Mean Difference
SMD: Standardized Mean Difference
OR: Odds Ratio
RR: Risk Ratio
CI: Confidence Interval
MD: Mean Difference
CBCT: Cone beam CT
