# Supplementary material for: The efficacy of anterior repositioning splints in the management of temporomandibular disc displacement: a systematic review and meta-analysis
Source: BMC Oral Health. 2025 Jul 28;25:1267. doi: 10.1186/s12903-025-06379-3 (PMC12302905; doi:10.1186/s12903-025-06379-3)
Supplement: Supplementary file 1 — Supplementary Material 1 [file 12903_2025_6379_MOESM1_ESM.docx]

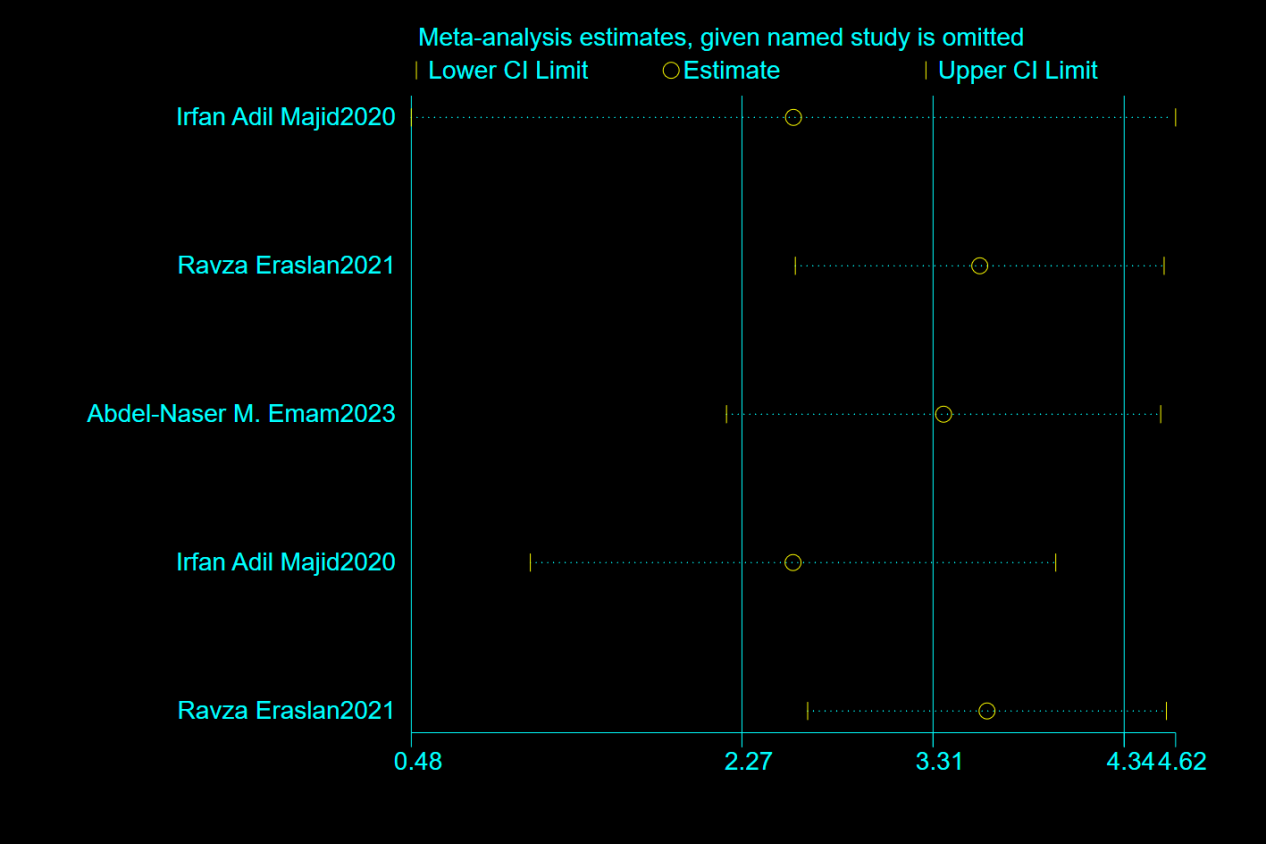


Figure S1 maximum active mouth opening( ARS group vs physical therapy group)sensitivity analysis


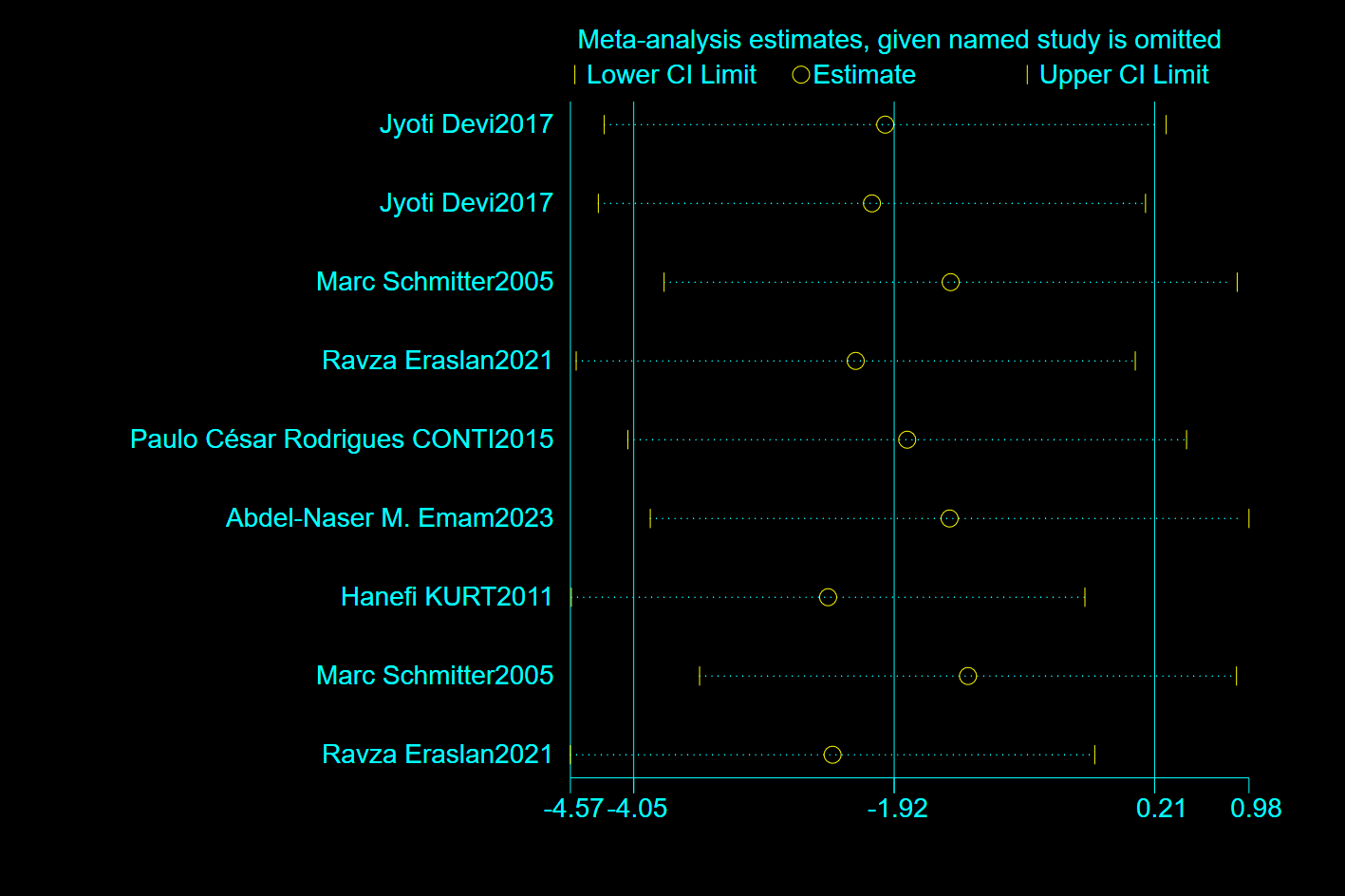


Figure S2 maximum active mouth opening(ARS group vs other occlusal splint therapy group)sensitivity analysis


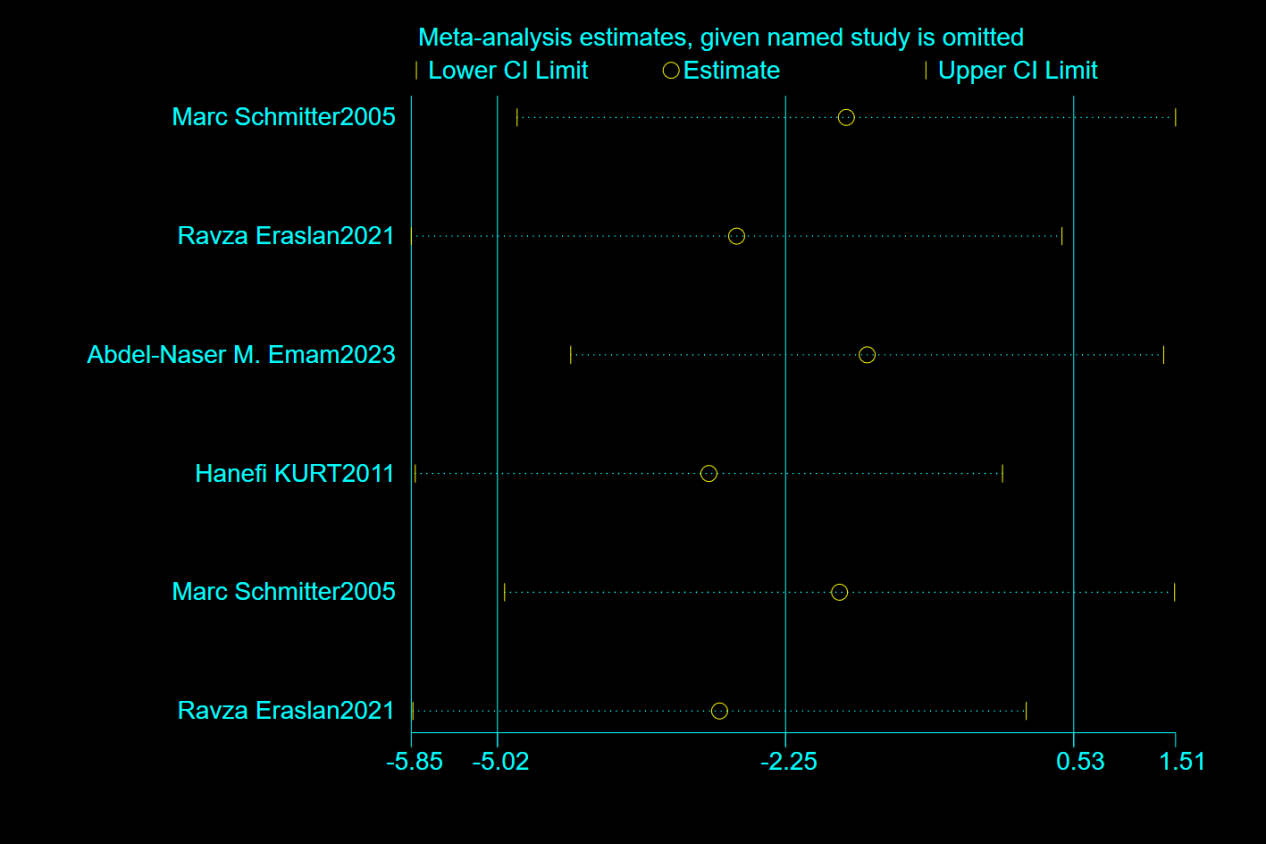


Figure S3 maximum passive mouth opening( ARS group vs other occlusal splint therapy group)sensitivity analysis


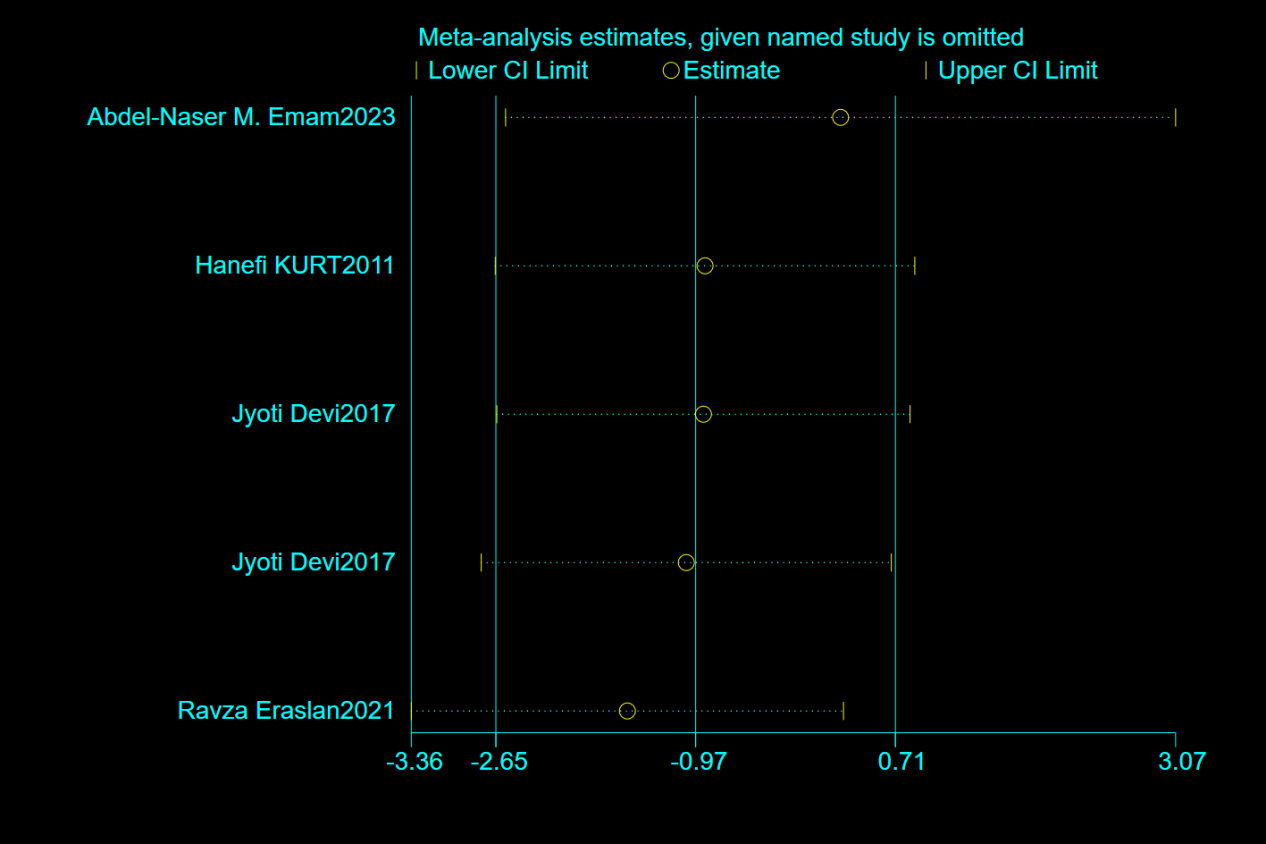


Figure S4 pain free mouth opening(ARS group vs other occlusal splint therapy group)sensitivity analysis


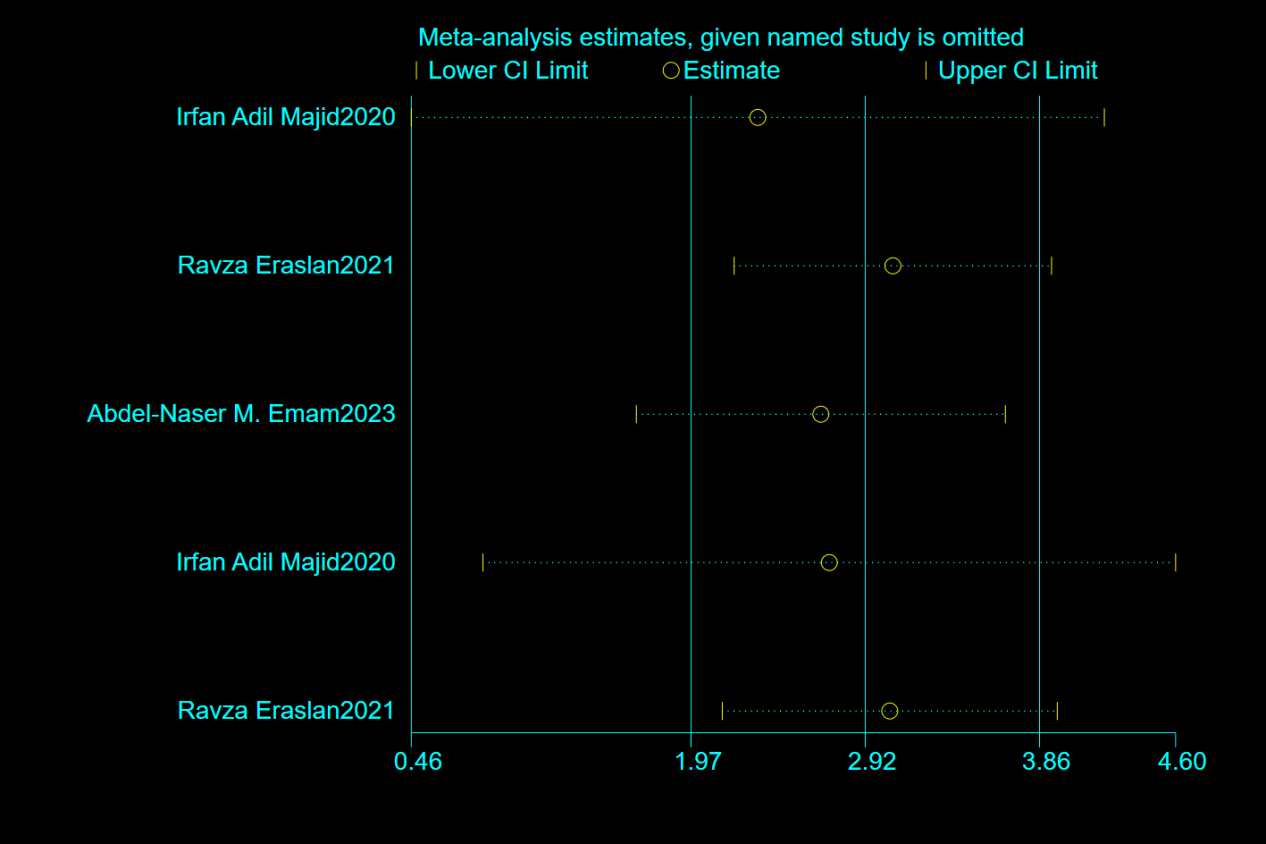


Figure S5 pain free mouth opening(ARS group vs physical therapy group)sensitivity analysis


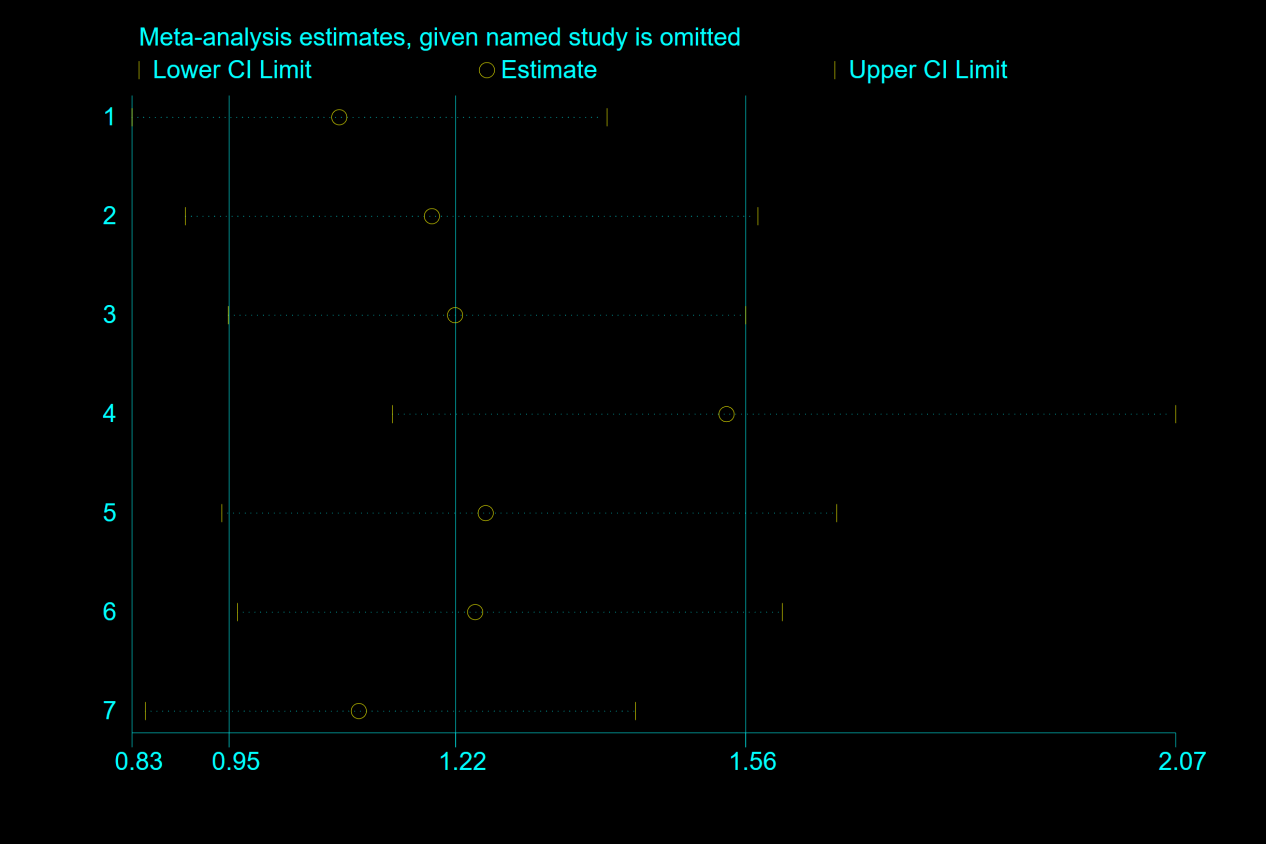


Figure S6 popping( ARS group vs other occlusal splint therapy group)sensitivity analysis


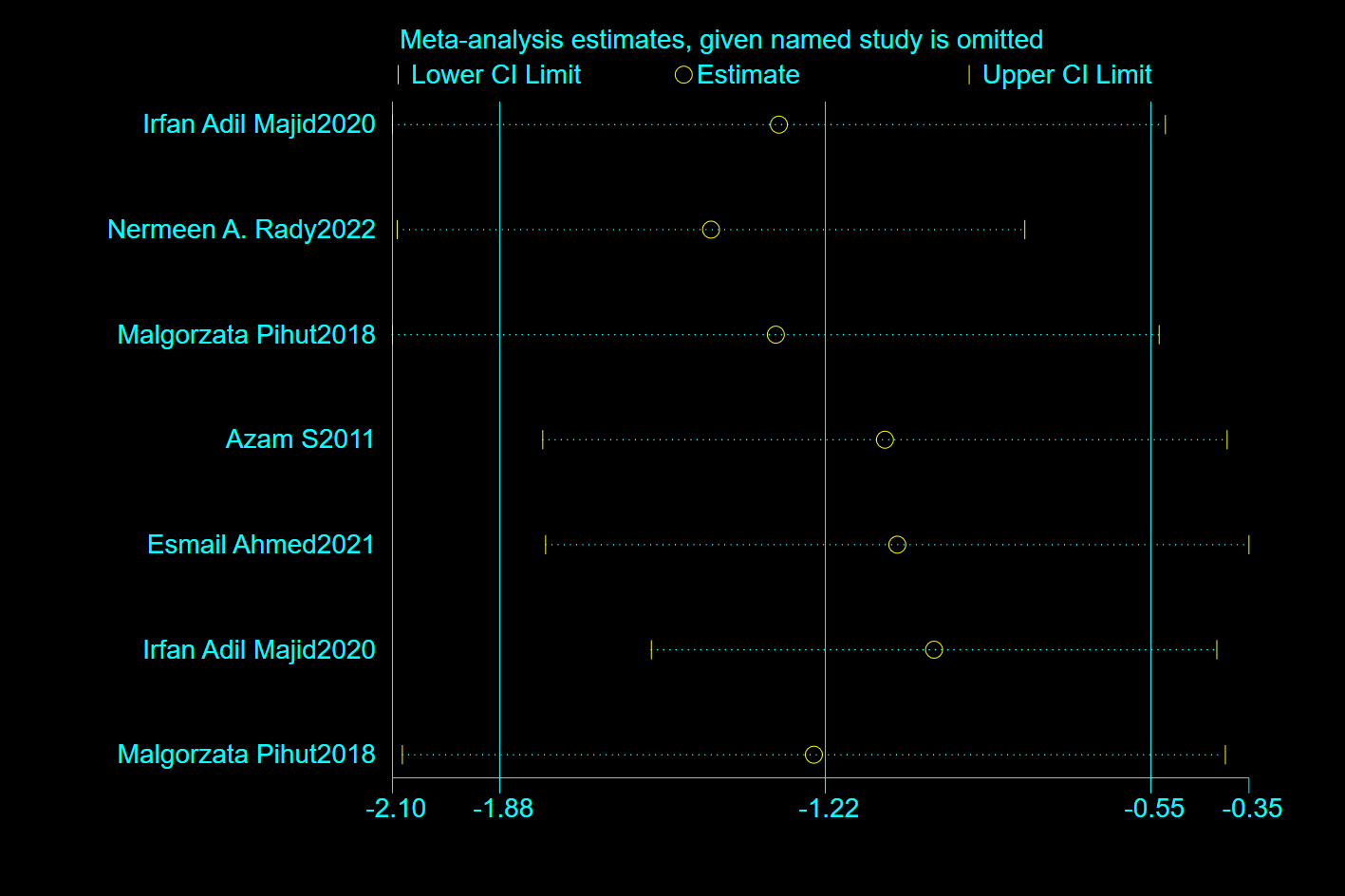


Figure S7 VAS( ARS group vs physical therapy group)sensitivity analysis


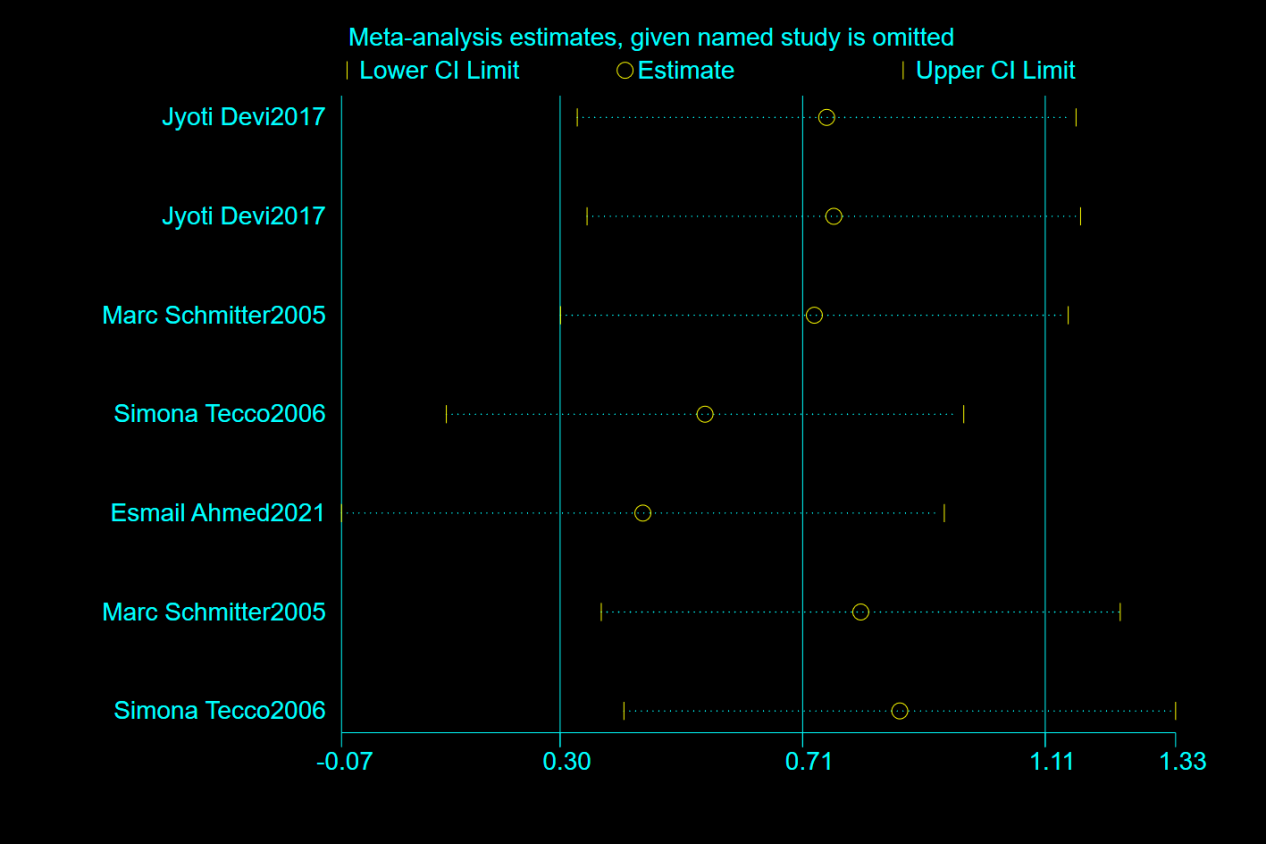


Figure S8 VAS(ARS group vs other occlusal splint therapy group)sensitivity analysis


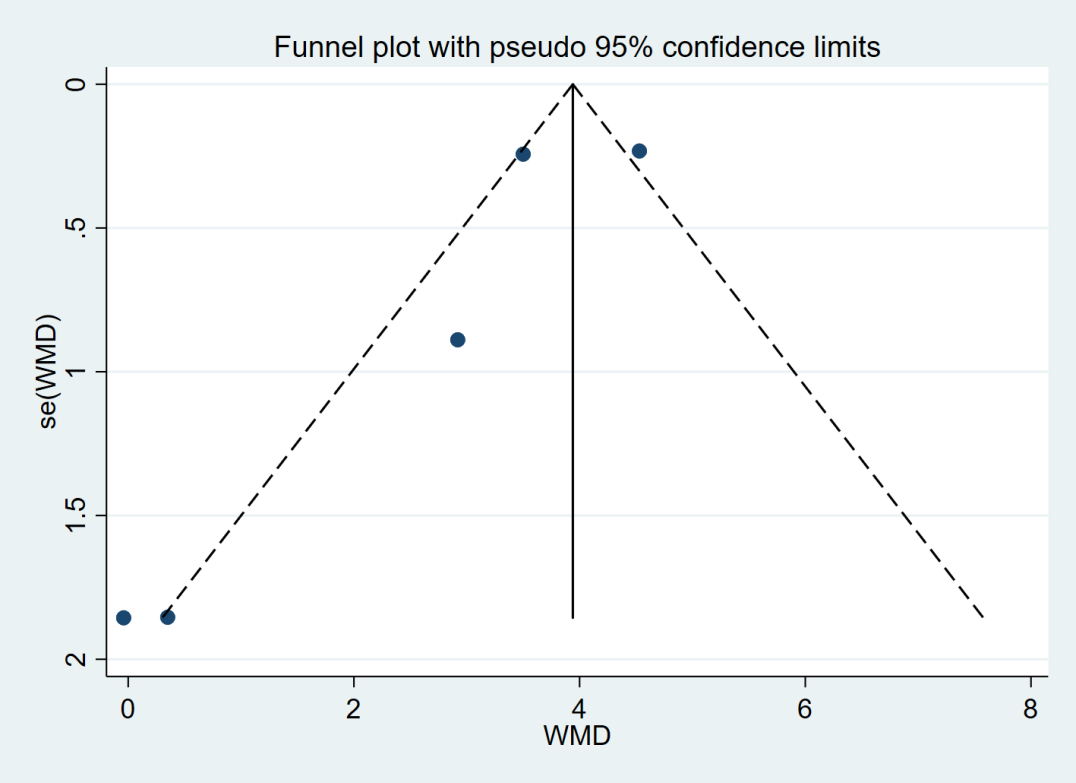


Figure S9 maximum active mouth opening( ARS group vs physical therapy group)funnel plot


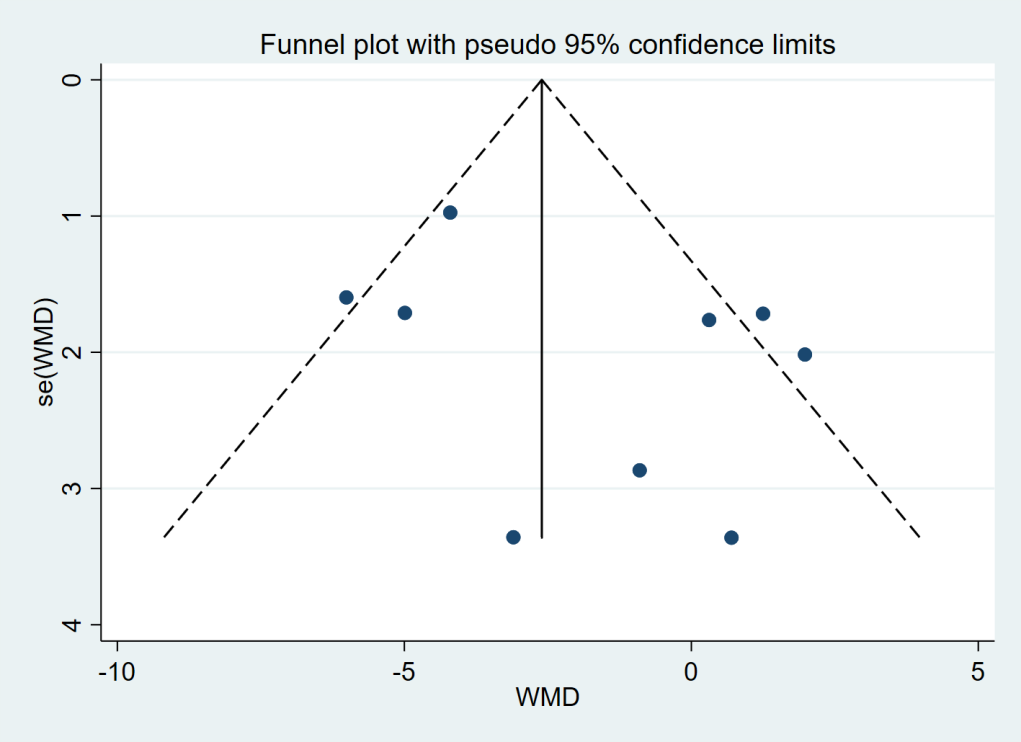


Figure S10 maximum active mouth opening(ARS group vs other occlusal splint therapy group)funnel plot


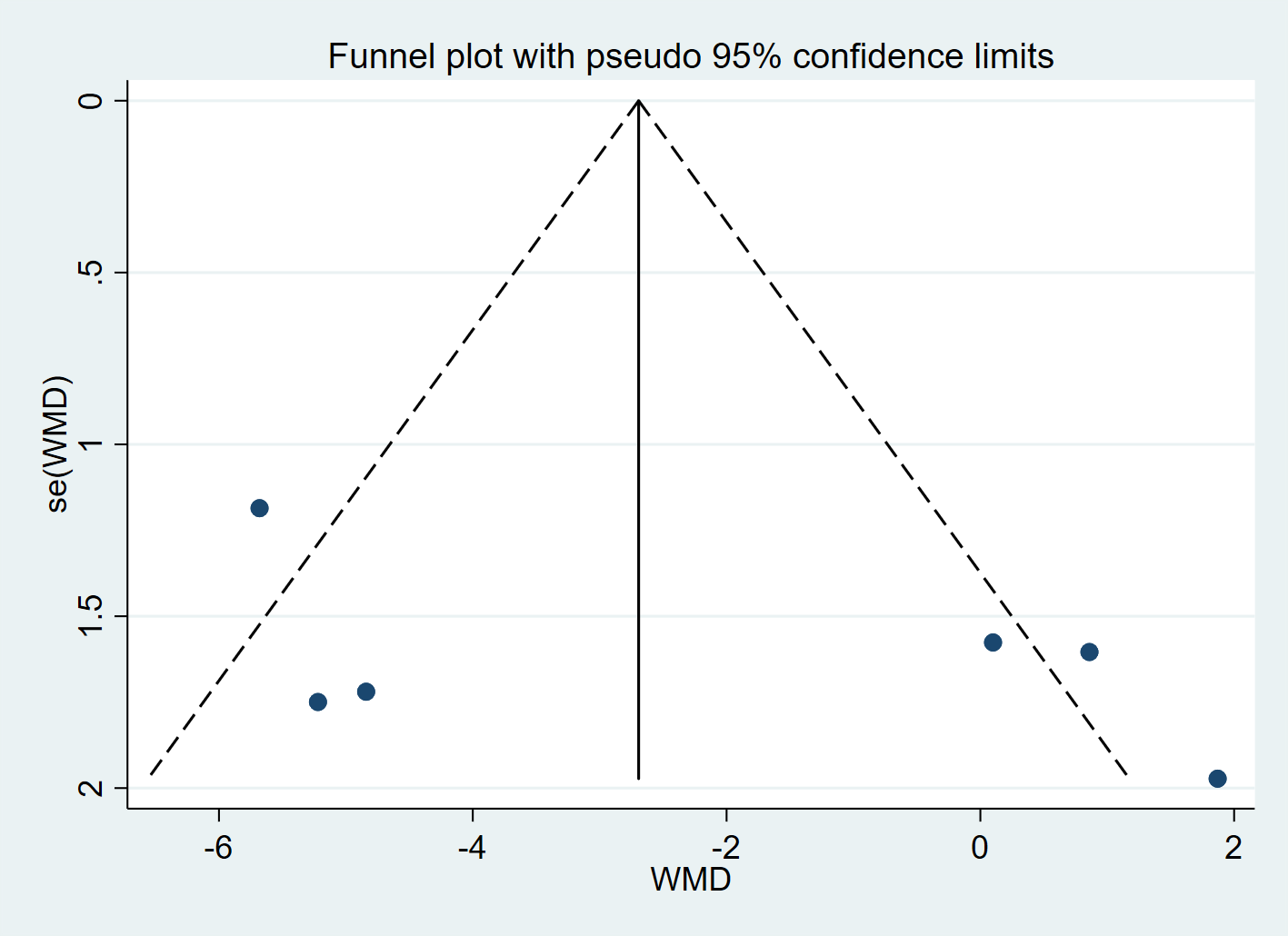


Figure S11 maximum passive mouth opening( ARS group vs other occlusal splint therapy group)funnel plot


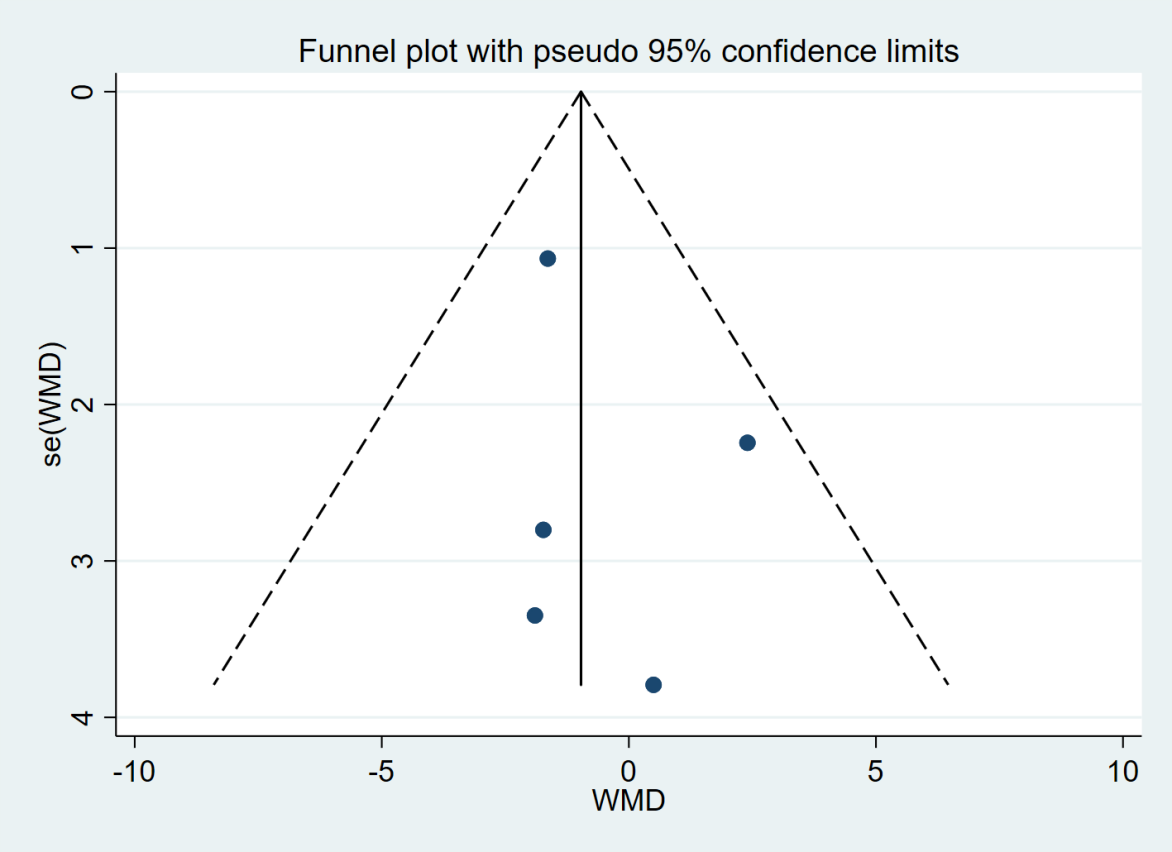


Figure S12 pain free mouth opening(ARS group vs other occlusal splint therapy group)funnel plot


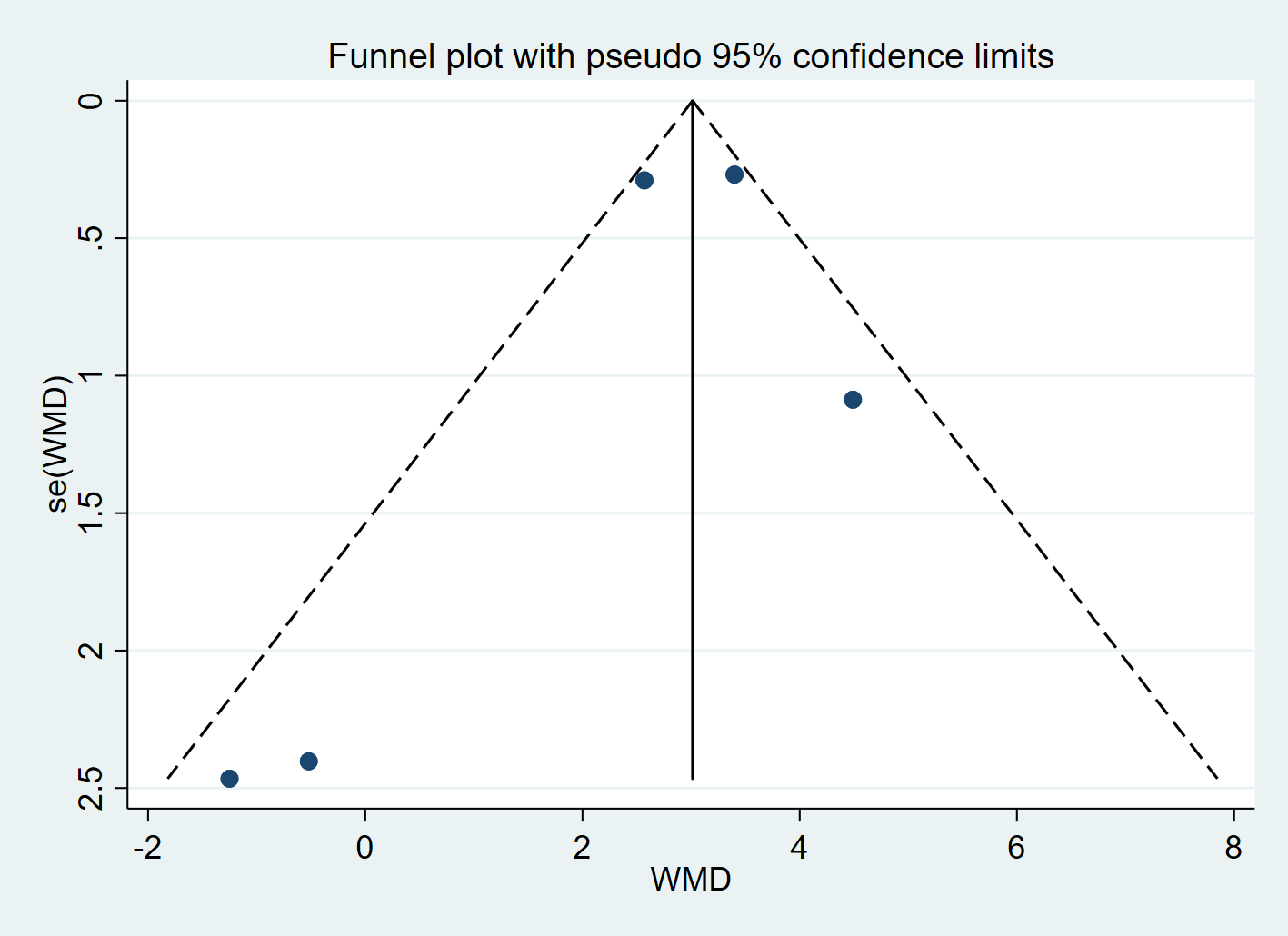


Figure S13 pain free mouth opening(ARS group vs physical therapy group)funnel plot


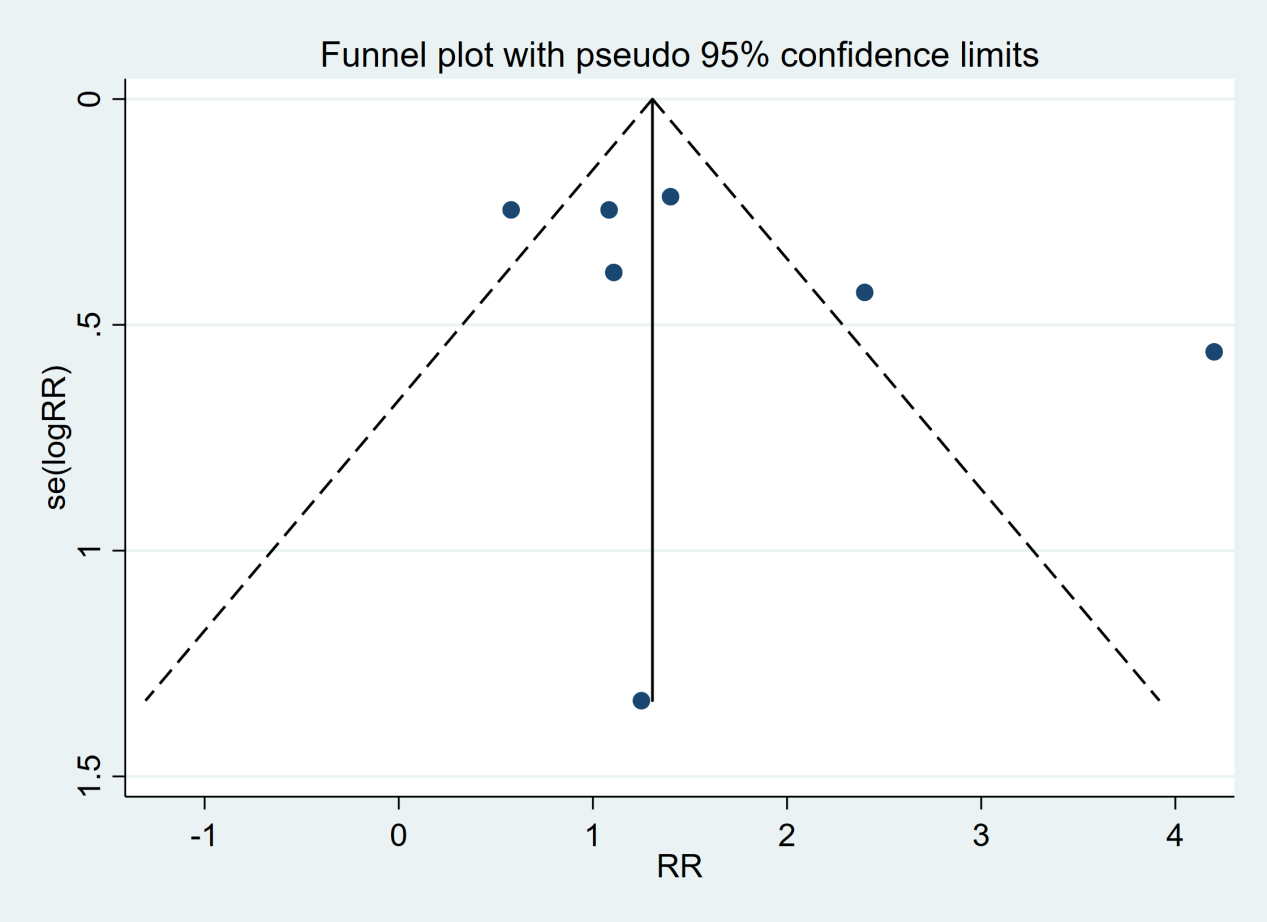


Figure S14 popping( ARS group vs other occlusal splint therapy group)funnel plot


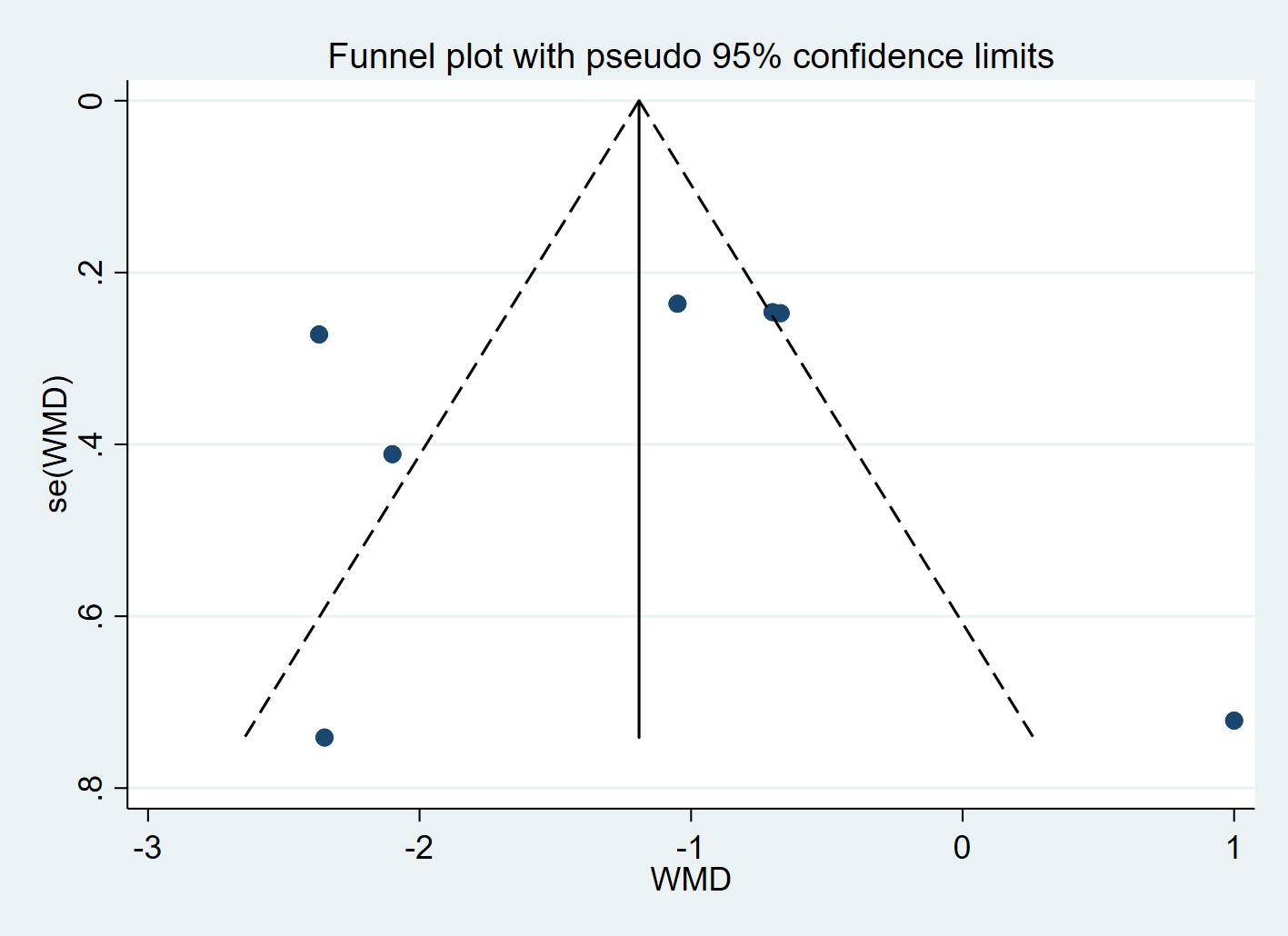


Figure S15 VAS( ARS group vs physical therapy group)funnel plot


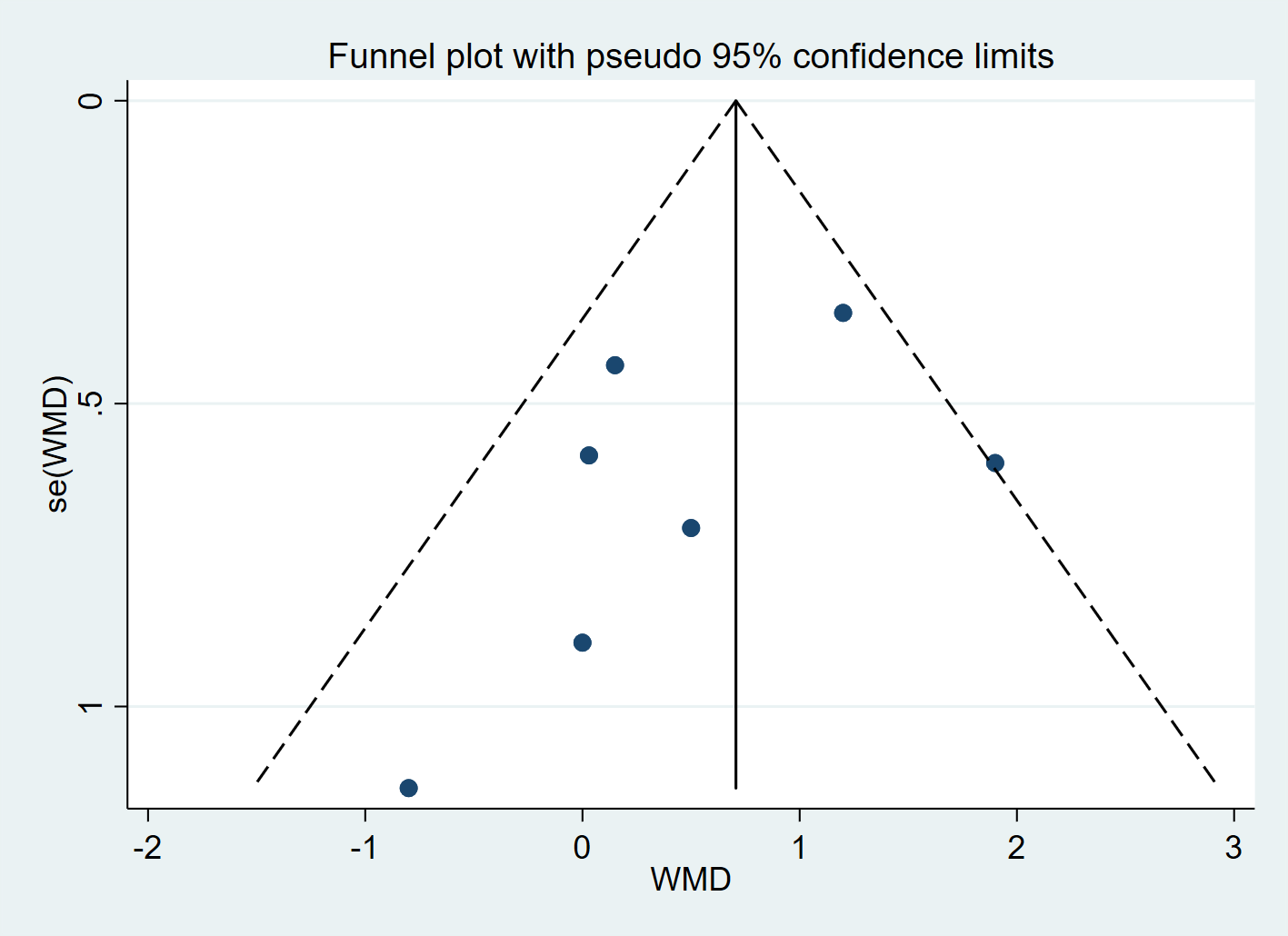


Figure S16 VAS(ARS group vs other occlusal splint therapy group)funnel plot
